# Janus-Faced Nature of Light in the Cold Acclimation Processes of Maize

**DOI:** 10.3389/fpls.2018.00850

**Published:** 2018-06-19

**Authors:** Gabriella Szalai, Imre Majláth, Magda Pál, Orsoly K. Gondor, Szabolcs Rudnóy, Csilla Oláh, Radomíra Vanková, Balázs Kalapos, Tibor Janda

**Affiliations:** ^1^Centre for Agricultural Research, Plant Physiology Department, Agricultural Institute, MTA, Martonvásár, Hungary; ^2^Department of Plant Physiology and Plant Molecular Biology, Eötvös Loránd University, Budapest, Hungary; ^3^Laboratory of Hormonal Regulations in Plants, Institute of Experimental Botany, Czech Academy of Sciences, Prague, Czechia

**Keywords:** abiotic stress, acclimation, chilling, gene expression, low temperature, photoinhibition, soluble sugars, *Zea mays* L

## Abstract

Exposure of plants to low temperature in the light may induce photoinhibitory stress symptoms, including oxidative damage. However, it is also known that light is a critical factor for the development of frost hardiness in cold tolerant plants. In the present work the effects of light during the cold acclimation period were studied in chilling-sensitive maize plants. Before exposure to chilling temperature at 5°C, plants were cold acclimated at non-lethal temperature (15°C) under different light conditions. Although exposure to relatively high light intensities during cold acclimation caused various stress symptoms, it also enhanced the effectiveness of acclimation processes to a subsequent severe cold stress. It seems that the photoinhibition induced by low temperature is a necessary evil for cold acclimation processes in plants. Greater accumulations of soluble sugars were also detected during hardening at relatively high light intensity. Certain stress responses were light-dependent not only in the leaves, but also in the roots. The comparison of the gene expression profiles based on a microarray study demonstrated that the light intensity is at least as important a factor as the temperature during the cold acclimation period. Differentially expressed genes were mainly involved in most of assimilation and metabolic pathways, namely photosynthetic light capture via the modification of chlorophyll biosynthesis and the dark reactions, carboxylic acid metabolism, cellular amino acid, porphyrin or glutathione metabolic processes, ribosome biogenesis and translation. Results revealed complex regulation mechanisms and interactions between cold and light signalling processes.

## Introduction

Low temperature is one of the most important factors limiting the spread and production of plants worldwide. This is especially true for field crops of tropical or subtropical origin. In the case of chilling-sensitive maize plants, temperatures in the 10–15°C range decrease the capacity for biomass production, while the exposure of plants to still lower temperatures for a prolonged period may lead to irreversible damage and the death of the plants (Bredenkamp and Baker, 1994; Greaves, 1996). Efficient early germination and growth at cool temperatures in the spring is a critical part of resistance to low temperature stress in young maize plants. One of the main aims of breeders is to develop cold-tolerant genotypes, which can be sown either earlier in order to extend the vegetation period, or in cooler geographical zones. Although chilling-sensitive plants are generally considered to lack the ability to develop chilling resistance when exposed to low but non-injurious temperatures, they can also be able to adapt to lower, usually non-freezing temperatures to some extent (Anderson et al., 1994; Prasad, 1996; Janda et al., 1998).

The mechanisms of cold acclimation are mainly studied in overwintering cereals, where a certain period of low, non-freezing temperature is necessary to achieve the maximum level of freezing tolerance even in the case of frost-tolerant winter varieties. This process is generally called frost hardening or cold hardening. Cold hardening is the result of various physical and biochemical processes, including the adjustment of membrane composition and the accumulation of certain protective substances, among them stabilising compounds such as polyamines, osmoprotectants or antioxidants (Winfield et al., 2010; Boldizsár et al., 2016a,b). The synthesis of these compounds and the regulation of acclimation mechanisms are mediated by a complex signal transduction network (Van Buskirk and Thomashow, 2006). However, it has long been known that light is necessary for the development of freezing tolerance as well as low temperature. Without enough light during the cold hardening period even winter cereals with a potentially high level of frost hardiness are incapable of achieving freezing tolerance (Gray et al., 1997; Apostol et al., 2006). Light has been shown to mediate the development of freezing tolerance via several biological processes. These include photosynthesis-related processes, the expression level of stress-related genes and the synthesis of various protective compounds (Janda et al., 2014).

A strong overlap has been shown between factors involved in sensing and transducing light and temperature in plants (Legris et al., 2016, 2017). Acclimation to low temperatures also respond to light and temperature signals (Catalá et al., 2011; Lee and Thomashow, 2012). In hardy plants light is a critical factor during the low temperature hardening period, but in chilling-sensitive plants light often shows another face during exposure to low temperature inducing photoinhibition, which contributes to the development of chilling symptoms in these plants (Gururani et al., 2015). Photoinhibition is a light-induced decline in photochemical activity and occurs when the light energy available exceeds the receptive capacity of the photosynthetic processes and the level that can be neutralised via different protective mechanisms (recently reviewed by Pospíšil, 2016). The mechanisms underlying the development of freezing tolerance during the hardening period of model plants, such as Arabidopsis, or cold-tolerant cereal species, such as wheat or oat, have been widely studied, but these results cannot be generalised for the very chilling-sensitive C4 plants. In recent years several cold-responsive genes have been identified in maize, but these studies were usually focused either on the comparison of genotypes with different levels of chilling tolerance (Sobkowiak et al., 2014, 2016; Li et al., 2016) or on the different phases of cold stress responses (Trzcinska-Danielewicz et al., 2009), and the role of light has not been discussed.

In the present work we hypothesised that light, in spite of its photoinhibitory effects, may also have a similar role in the cold acclimation processes in chilling sensitive plants, similarly as it was found in cold tolerant winter cereals or in *Arabidopsis*. Experiments were designed in order to characterise the contribution of light during the cold acclimation period to the development of a certain level of cold tolerance in maize plants. A detailed microarray study was also carried out, and certain physiological, biochemical and genetic factors contributing to cold tolerance were tested. Results may point out the possible involvement of low temperature-induced photoinhibition in the development of cold tolerance as a signal during the cold acclimation period in chilling sensitive plants.

## Materials and methods

### Plant material and growth conditions

In the 1st set of experiments seeds of maize plants (*Zea mays* L. hybrid Norma) were germinated between wet filterpapers for 3 days then grown in a modified Hoagland solution (Pál et al., 2005) for a week at 22/20°C at a photosynthetic photon flux density (PPFD) of 180 μmol m^−2^ s^−1^ (growth light 1; GL1) with 16/8 h light/dark periodicity (control plants). The plants were then hardened at 15/13°C for 3 days under three different light conditions: GL1; low light intensity 1 (LL1): 46 μmol m^−2^ s^−1^; and low light intensity2 (LL2): 14 μmol m^−2^ s^−1^). After this the plants were transferred to 5°C at continuous GL1 for 3 days, and then back to 22/20°C for a 1-day recovery period. Leaf and root samples were collected from treated plants after the acclimation (13 day-old-plants), chilling (16 day-old-plants) and recovery (17 day-old-plants) periods for the estimation of viability and oxidative stress, and for carbohydrate analysis.

In the second set of experiments maize plants were grown for 11 days after 3 days germination at 22/20°C at higher PPFD, (growth light 2, GL2 = 387 μmol m^−2^ s^−1^) with 16/8 h light/dark periodicity (control plants). Some of the plants were then cold acclimated at 15/13°C for 3 days either at GL2 or at moderately low light intensity 3 (LL3): 283 μmol m^−2^ s^−1^; or at moderately low light intensity 4 (LL4): 107 μmol m^−2^ s^−1^. Afterwards all the plants were transferred to 5°C at continuous growth light (GL2) for 3 days, followed by a 7-day recovery period at 22°C. Samples were collected for biochemical analysis from the control plants, and from treated plants after the acclimation (17 day-old-plants), chilling (18 and 20 day-old-plants) and recovery periods (21, 24, and 27 day-old-plants).

### Estimation of lipid peroxidation

The lipid peroxidation analysis was based on the measurement of the malondialdehyde (MDA) level according to Gondor et al. (2016a) using 0.5 g of 3rd leaves of the plants. Five replicates were measured from each treatment and at least three leaves were used for one replicate.

### Electrolyte leakage test

Two leaf discs (8 mm diameter) cut from the middle part of the 3rd leaves were used for the determination of membrane damage. The measurements were carried out according to Szalai et al. (1996). Ten replicates were measured from each treatment and one leaf was used for one replicate.

### Determination of chlorophyll content

The total chlorophyll content was measured on the 3rd leaves using a SPAD-502 chlorophyll meter (Minolta Camera Co., Ltd, Japan) as described by Pál et al. (2011). At least 30 replicates were measured from each treatment and one leaf was used for one replicate.

### Measurement of chlorophyll-a fluorescence

Chlorophyll-*a* fluorescence induction was analyzed using a PAM fluorometer with a LED-Array Illumination Unit IMAG-MAX/L (λ = 450 nm) (Imaging-PAM, Walz, Germany). After 30-min dark adaptation, quenching analysis was carried out on the leaves at laboratory temperature using 250 μmol m^−2^ s^−1^ actinic light intensity until the steady state level of photosynthesis was reached (15 min). PPFD for the saturation pulses was higher than 2,000 μmol m^−2^ s^−1^. The fluorescence induction parameters were calculated as described by Klughammer and Schreiber (2008). Ten replicates were measured from each treatment and one leaf was used for one replicate.

### Soluble sugar analysis

0.5 g of leaf and root samples were measured according to Gondor et al. (2016b). Five replicates were measured from each treatment and at least three leaves were used for one replicate.

### Microarray analysis

Three biological replicates each with three technical replicates (each consisting of seven plants) for the microarray analysis were harvested from the 3rd leaves of maize plants. Samples were collected from the control and the cold acclimated plants from the 2nd experiment. Two hundred ng of the total RNA was extracted using an RNEasy Plant Mini Kit (Qiagen, Hilden, Germany) and pooled from the three technical replicates and used for cRNA amplification. The quality of the extracted RNA was examined using the Agilent 2100 Bioanalyzer (Agilent Technologies Inc., Palo Alto, CA). The samples were labelled with Cy3 (Low Input QuickAmp, Agilent), and 1,650 ng cRNA was hybridized to the Agilent Whole Corn Gene Expression Microarray 4 × 44K chip according to the manufacturer's instructions (Agilent). The array was scanned using an Agilent Scanner, Extended Dynamic Range (100% and 10% laser intensities, 5 micron resolution). Signal intensities and normalization processes were detected with the Agilent Feature Extraction (FE 9.5) and GeneSpring (Agilent) Softwares. The fold change (FC) values of the samples were compared for Control vs. GL2, Control vs. LL3, Control vs. LL4, GL2 vs. LL3, GL2 vs. LL4 and LL3 vs. LL4 in a simple loop design. Genes with logFC > |2| and P < 0.01 were considered as differentially expressed. The functional annotation of the probe sequences was performed in the *Zea mays* taxon and using the BLASTX with the default settings (Altschul et al., 1997).

### Validation of microarray results using qRT-PCR

The microarray analysis was validated by qRT-PCR, using the same total RNA samples for microarray analysis and cDNA synthesis. cDNA was synthesized from 500 ng RNA with a RevertAid™ First Strand cDNA Synthesis Kit (Thermo Fisher Scientific, Waltham, USA). Seven genes that showed significant changes in the microarray analysis and represented different functional categories in response to cold treatment under different light conditions, namely NM214, LOC285, BT062987, BT060766, BT035304, XM587-2, and LOC531 were chosen for the validation. The gene expression changes were examined using an ABI StepOnePlus Real-Time PCR System (Thermo Fisher Scientific) with Maxima SYBR Green/ROX qPCR Master Mix (Thermo Fisher Scientific). The two internal control genes used for normalizing the variations in cDNA amounts were *actin* and the membrane protein gene *PB1A10.07c* (*MEP*). The geometric mean of the internal control data was applied for normalization. The relative changes in gene expression were compared to the control group and quantified according to the comparative CT method (2^−ΔΔCT^ method, Livak and Schmittgen, 2001). All seven genes showed similar expression patterns with quantitative real-time PCR and microarray analysis, demonstrating the reliability of the microarray results (Figure S1). Although the fold-change values determined by microarray analysis and RT-PCR were slightly different, the microarray values were generally lower than the qRT-PCR data. This might have been due to technical differences in the methods used for analysis and normalization (Liu et al., 2013).

### Venn-diagram analysis

Lists of up- and downregulated genes (Control vs. GL2, Control vs. LL3, Control vs. LL4, GL2 vs. LL3, GL2 vs. LL4 and LL3 vs. LL4) with *e*-value < 1^−4^ were compared in order to reveal the uniqueness and overlap of individual genes using the InteractiVenn toolkit (Heberle et al., 2015).

### Principal component analysis (PCA)

The similarity of the gene expression data sets for each comparison was analyzed based on the significant logFC values (*P* < 0.01) of array probes using a *var-covar* matrix to principal component analysis (Hammer et al., 2001).

### GO analysis

The EST hits of the significantly differentially expressed Agilent probes, showing *P* < 0.01, were matched to their Affymetrix IDs using the bioDBnet webtool (Mudunuri et al., 2009) which were used as input of SEA (Singular enrichment analysis) carried out using the agriGO v2.0 web-based toolkit (Tian et al., 2017). The maize locus ID v3.30 (Gramene Release 50) as a reference. Ranking scores between GO nodes, a hypergeometric statistical test was carried out on the *p*-values to correct them for multiple testing errors taking into account the false discovery rate (FDR) (Benjamini and Yekutieli, 2001). GO nodes with *q* < 0.05, where q means the multiple test adjusted *p*-value, were considered as overrepresented term.

### Metabolic pathway analysis

In order to classify and display genes into metabolic pathway groups, MapMan software (Thimm et al., 2004) was used. The maize mapping file was developed as follows. The EST hits of the significantly differentially expressed Agilent probes, showing *P* < 0.01, were matched to their Affymetrix IDs using the bioDBnet webtool (Mudunuri et al., 2009). The maize Affymetrix chromosomal locations was taken as template for mapping the Affymetrix IDs of the treatments to metabolic pathway groups.

### Statistical analysis

The experiments were repeated 3 times and representative data are shown. The results were the means of at least 5 measurements. The data were statistically evaluated using the standard deviation, ANOVA and *t*-test methods.

## Results

In order to characterise the role of light during the cold acclimation period in maize plants, 2 set of experiments were designed. One with relatively low growth and hardening light intensities, and a 2nd one with relatively higher light conditions, where the possible role of photoinhibition is more dominant.

### 1st experiment: cold hardening at relatively low light intensity

**Figure 1:**
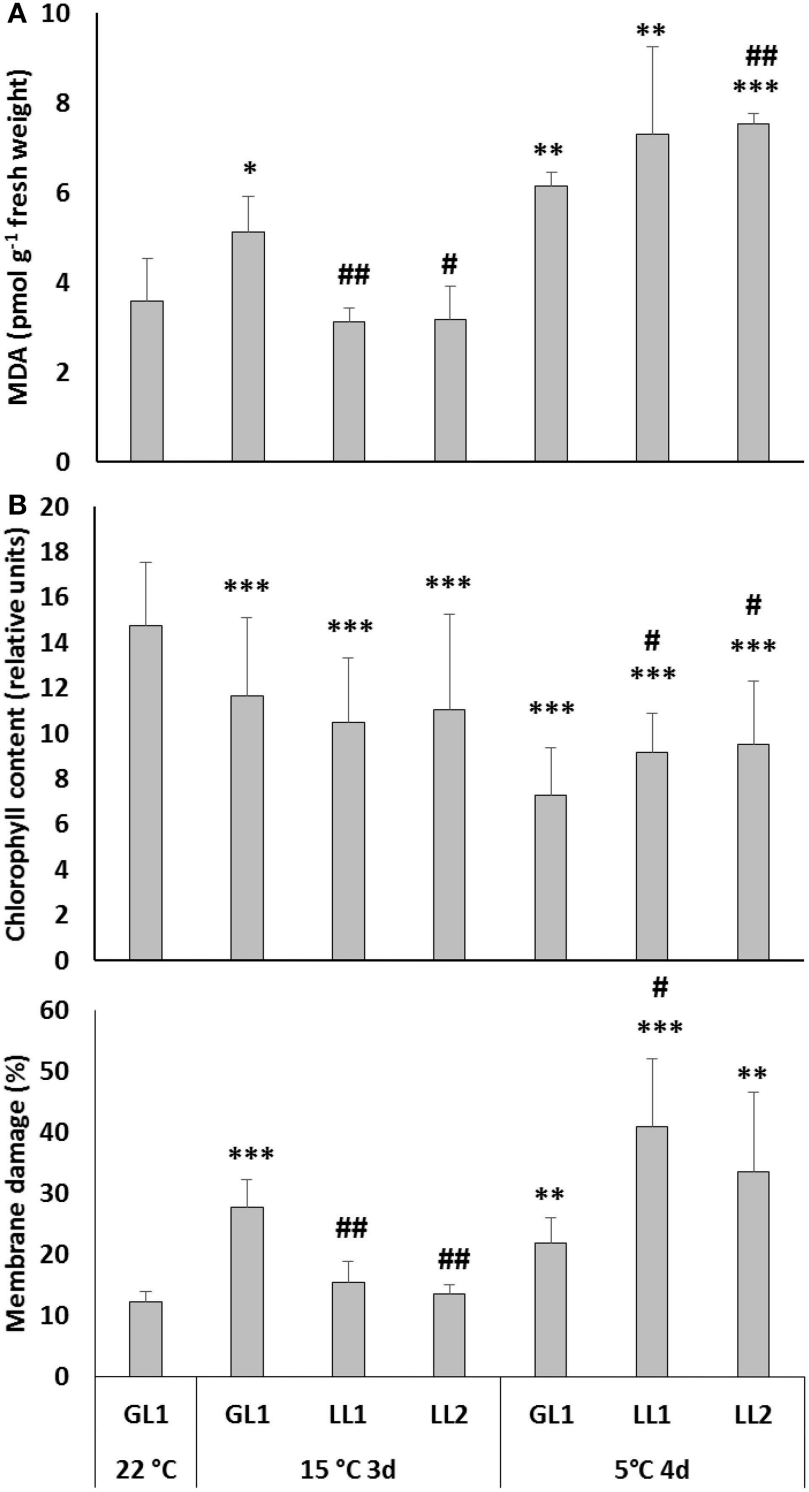
MDA levels **(A)** chlorophyll content represented with SPAD-values **(B)** and membrane damage measured as electrolyte leakage **(C)** in the leaves of plants grown at GL1 before and after cold hardening at 15°C at GL1, LL1, or LL2, and after chilling stress at 5°C at GL1. ^*^, ^**^, ^***^Significant differences compared to the GL1 22°C plants at the *p* < 0.05, 0.01, and 0.001 levels, respectively. ^#^, ^##^, Significant differences compared to the GL1 plants on the same day at the *p* < 0.05 and 0.01 levels (mean ± SD, *n* = 5 for MDA, and *n* = 10 for SAPD and electrolyte leakage values).

In the 1st set of experiments maize plants were grown at relatively low light intensity (GL1 = 180 μmol m^−2^ s^−1^), after which some plants were hardened at even lower light intensity (LL1 or LL2), followed by chilling stress under continuous illumination at GL1. The MDA level was measured to detect the extent of oxidative stress and was found to increase during hardening at GL1 in both leaves (Figure 1A) and roots (Figure S2), but did not change at LL1 or LL2. During chilling at GL1 the amount of MDA did not change significantly in the leaves but substantially increased in the roots. In the leaves of plants which were acclimated at low light intensity the chilling-induced increase in the MDA level was more pronounced. Interestingly, the light intensity during the hardening period also affected the chilling-induced MDA levels in the roots, where the lower the PPFD, the lower the MDA level detected (Figure S2). This means that not only the leaves, but also the roots exhibited light-dependent cold responses.

The effect of light during the hardening period was also monitored by estimating the chlorophyll content. The SPAD values showed that although hardening in the light provided protection against subsequent cold-induced oxidative stress in the leaves (Figure 1B), the chlorophyll content was slightly higher in plants hardened at lower light intensity.

These results suggest that although light may reduce the chilling-induced oxidative stress in maize, it may also have negative impacts on certain physiological processes indicated by the SPAD and MDA values; and probably photooxidative damage also occurred. Furthermore, loss of photosynthetic pigments may be an adaptive response required to balance the capacity for photosynthetic electron transport with the rate of metabolism. This may also decreased membrane damage in GL1 plants. Therefore the evaluation of the effect of light during cold acclimation in maize was continued by measuring electrolyte leakage. Cold acclimation at GL1 slightly increased the electrolyte leakage from leaf cells (Figure 1C). However, after exposure to severe stress at 5°C this increase was more pronounced in plants acclimated at LL1 and LL2.

### 2nd experiment: cold hardening under medium light conditions

Since, despite its beneficial effects during the cold acclimation period, light may also induce secondary stress (photoinhibition), the 2nd set of hardening experiments was performed at higher light intensities. In this set of experiments plants were originally grown at higher PPFD (GL2 = 387 μmol m^−2^ s^−1^) after the germination period. Since plants were generally adapted to this higher light during their growth period, neither cold acclimation nor chilling at 5°C for 3 days caused substantial decrease in the chlorophyll content estimated with SPAD values (data not shown). However, visual analysis showed that the post-chilling symptoms during the recovery period (4 days at 22°C after 3-day cold treatment at 5°C), such as chlorosis in the distal parts of the leaves, were the most pronounced in un-acclimated plants, being less obvious in plants acclimated at higher light intensities (Figure S3).

**Figure 2:**
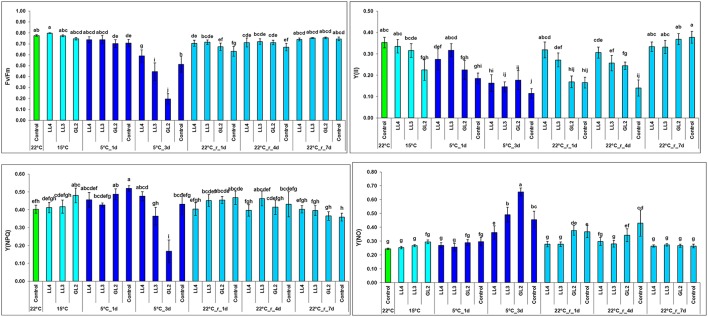
Effect of cold hardening in maize at 15°C under different light conditions, followed by chilling stress at 5°C and 7-d recovery on the chlorophyll-a fluorescence induction parameters Fv/Fm, Y(II) (the quantum yield of Photosystem 2 estimated as ΔF/Fm′), Y(NPQ) and Y(NO) (the regulated and non-regulated non-photochemical quenching). Different letters on the bars represent statistically significant differences at *p* < 0.05.

The chlorophyll-a fluorescence induction technique provides a valuable tool to detect changes in Photosystem 2, especially under photoinhibitory conditions (Foyer et al., 2017). Fv/Fm, which represents the maximum quantum efficiency of Photosystem 2, did not change significantly after growth at hardening temperature. However, it substantially decreased in a light-dependent manner at 5°C in all the plants. Interestingly, this decrease was more pronounced in plants acclimated at higher light intensities. The decrease in Fv/Fm in non-acclimated plants exhibited values intermediate to those of plants acclimated at GL2 and LL (Figure 2). It seems that the hardening period preconditioned the plants for the subsequent photoinhibitory effect of low temperature. The recovery of Fv/Fm was relatively fast. The quantum yield of Photosystem 2, Y(II), decreased within 3 days at 15°C at GL2. However, at 5°C Y(II) was not better in the non-hardened plants than in those hardened at GL2, and recovery was also slower than in acclimated plants (Figure 2). The negative impact of relatively high light intensity on the quantum yield was still detected during the beginning of the recovery period, but the decrease in this parameter was reversible within 7 days. The most significant changes in the regulated non-photochemical quenching, Y(NPQ), could be found in plants acclimated at GL2, where this parameter slightly increased; however, Y(NPQ) substantially decreased in the same plants after exposure to 5°C for 3 days. This parameter recovered rapidly (within 1 day) at 22°C. Non-regulated non-photochemical quenching, Y(NO), showed a similar pattern but with the opposite trend, substantially increasing in a light-dependent manner at 5°C, especially in plants acclimated at GL2.

In contrast to the 1st set of experiments, where the light intensity was relatively low, the MDA content did not change substantially in the leaves in the 2nd set of experiments (Figure S4). A notable increase could only be detected after 4 days of recovery, probably due to the post-chilling effect. However, as in the previous experiment, MDA showed a light dependent response in the roots, increasing even during the hardening period, especially at higher light intensity. Recovery was also light-dependent.

### Alteration in gene expression profiles at low temperature

In order to obtain a better understanding of the molecular mechanisms underlying the role of light during the cold acclimation period in maize, gene expression analysis was performed using the microarray technique. Samples were taken from control plants growing at GL2 at 22°C and from plants hardened for 3 days at 15°C at GL2, LL3, or LL4.

In total, 42,050 transcripts were examined on the Agilent chip. Genes were considered to have significantly different expression with logFC > |2| and the *P*-value was < 0.01, which resulted 677, 467, 1,780, 63, 1,317, and 1,240 individual significant array probes in Control vs. GL2, Control vs. LL3, Control vs. LL4, GL2 vs. LL3, GL2 vs. LL4, and LL3 vs. LL4, respectively (Table S1). After the functional annotation of the probe sequences with a *e*-value of < 1e^−4^ cutoff, the results showed that although, as expected, low temperature up- or down-regulated the expression of hundreds of genes, light also substantially altered the expression level of a similar number of genes (Table S1). The Venn diagram comparison can be seen on Figure S5. Selected genes related to photosynthesis with logFC > |2| and known function (*e*-value < 1^−4^) that were differentially expressed in Control vs. GL2 or GL2 vs. LL4 are listed in Table S2.

### Cluster analysis

**Figure 3:**
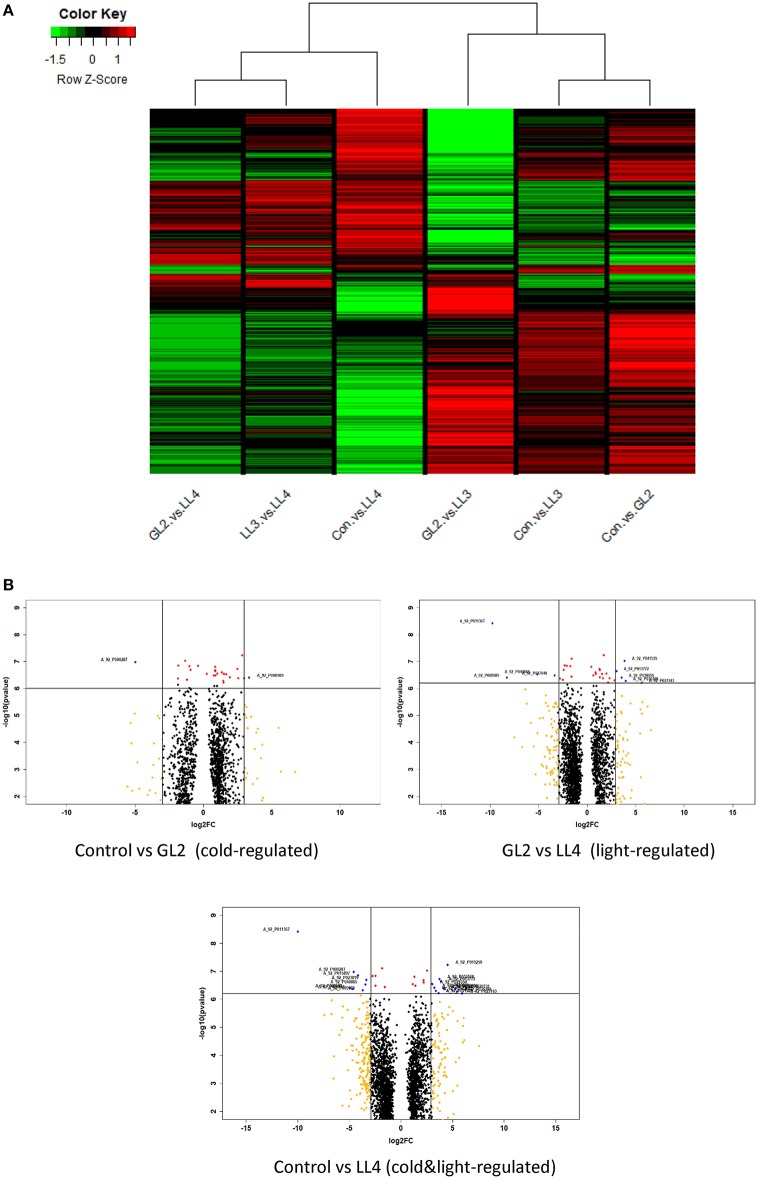
**(A)** Cluster heat map of cold-regulated (Control vs. GL2), Control vs. LL3, Control vs. LL4 (double factor-regulated) and GL2 vs. LL3, GL2 vs. LL4, LL3 vs. LL4 (light-regulated) comparisons with log2FC < −0.1 and log2FC > 0.1. Red colour represents up-regulated expression and green colour represents down-regulated expression. Each row represents a differentially expressed gene function pathway; **(B)** Volcano plots of three comparisons indicating either cold, light, and their combined regulation. Genes with high level of expression and significance were emphasized (filter red dots indicate if *p* < 0.0001, orange if log2FC > |3| and blue if both).

To understand the relationships and discrepancy of the effect of cold, light or their combination more comprehensively, cluster analysis was carried out according to log2FC values of significantly differentially expressed genes of all comparisons. The same type of regulations was gathered in clusters with similar gene expression profiles. The cluster of the columns of the heat map is shown in Figure 3A. The results indicate that effect of different illuminations clearly distinguished. Low light intensity LL4 induced the expression significantly expressed genes at different levels both at normal and low temperatures. Other light intensities, GL2 and LL3 had altered effect on genes investigated.

### Differential expression analysis

Volcano plotscarried out on the significantly expressed microarray probes of each comparisons which either regulated by cold or light or both factors (Figure 3B). Genes with log2FC > |3| and *p* < 0.0001 were filtered and considered as the most outlier gene in each regulations. The biological functions were annotated and listed in Table S3. The highest number of genes high log2FC and low *p*-value were found of the effect of LL4 in the cold and most of them were up-regulated, such as the acyclic sesquiterpene synthase which was expressed alone under these conditions. Regarding genes with known function, glutaredoxin-C13 was induced cold under GL2 and repressed by the LL3 and LL4.

### Principal component analysis

A scatter plot diagram and the loadings of the original variables were used to show the similarity between the global gene expression profiles of each comparison (Control vs. GL2, Control vs. LL3, Control vs. LL4, GL2 vs. LL3, GL2 vs. LL4, LL3 vs. LL4) (Figure S6). The biplot representation showed that the Control vs. GL2 and Control vs. LL3 comparisons were similar to each other, but differed from GL2 vs. LL3 in the expression profile along axis 1 (PC 1). The expression pattern of LL3 vs. LL4 was similar to that of GL2 vs. LL4 along axis 2 (PC 2). Control vs. LL4 was quite dissimilar to the other comparisons. The results suggested that illumination levels GL2 and LL3 had a different effect on gene expression than the LL4 illumination level.

### GO analysis

The microarray output was mapped for gene functions using the GO classification for genes differentially regulated at low temperature under different light conditions (Figures S7–S14). The GO enrichment analysis focused both on the effect of cold under GL2 and how the GL2 acts on global gene expression in contrast to LL4 in the cold.

Low temperature greatly influenced the global gene expression pattern under growth light (GL2) as compared to the control temperature (Figures S7, S8). Most of the genes significantly differentially up-regulated (*q* < 0.05) in the cold are originated from the cytoplasm (GO: 0005737), cytoplasmic membrane-bounded vesicles (GO: 0016023) the plastids (GO: 0009536) or in the mitochondria (GO: 0005739). The result of the ontology analysis of gene functions regulated by cold showed the over-representation of ribosome biogenesis (GO: 0042254), hexose biosynthetic (GO: 0046364) and aspartate and serine family amino acid metabolic processes (GO: 000258, GO: 0009069).

GL2 caused different gene expression as compared to LL4 in the cold in the GL2 vs. LL4 comparison (Figures S9–S11). Genes regulated by GL2 mostly expressed in intracellular membrane-bounded organelles (GO: 0043231) such as plastids (GO: 0009536) and the chloroplast (GO: 0009507). Many genes were also expressed in the cytoplasmic membrane-bounded vesicles (GO: 0016023). The following biological were greatly emphasized under GL2 illumination in the cold, such as chlorophyll biosynthesis (GO: 0015995), cellular nitrogen compound biosynthetic process (GO: 0044271), cellular amino acid biosynthetic process (GO: 0008652), ribosome biogenesis (GO: 0042254). Genes were also associated with the cellular nitrogen compound metabolic process (GO: 0034641) and consequently, cellular amino acid metabolic processes (GO: 0006520). Hexose and galactose biosynthetic processes (GO: 0019319, GO: 0006012) were also significantly over-represented under these conditions. Amongst molecular functions, glutathione transferase activity (GO: 0004364) and rRNA binding (GO: 0019843) were over-represented.

Double factor-regulation, i.e., low light (LL4) illumination in the cold caused higher alterations in the global gene expression than GL2 did that in the cold (Figures S12–S14). Genes functions were significantly over-represented of the effect of LL4 in the following cellular components, namely intracellular membrane-bounded organelles (GO: 0043231) and intracellular membrane-bounded organelles (GO: 0043231), such as mitochondria (GO: 0005739) and plastids (GO: 0009536), as well as cytoplasmic membrane-bounded vesicle (GO: 0016023). Gene functions were highlighted under this light regime are related to the cellular amino acid metabolic processes (GO: 0006520), especially serine amino acid metabolism. On the other hand, gene functions can be mapped to carboxylic acid metabolism (GO: 0019752), gluconeogenesis (GO: 0006094) and galactose metabolic process (GO: 0006012). Secondary metabolism (GO: 0019748) and coenzyme metabolism (GO: 0006732) were less affected. In general, most of the important biological processes of the cell were influenced in Control vs. LL4 comparison. The ribosome biogenesis (GO: 0042254) and consequently, the translation (GO: 0006412) and its regulation (GO: 0006417) were affected. Photosynthesis (GO: 0015979) was also affected by LL4 in the cold. Both the process of light capture via the modification of chlorophyll biosynthesis (GO: 0015995) and the dark reactions (GO: 0019685) changed. The amino acid metabolism were significantly affected, such as serine and glutamine metabolism. The genes in the cofactor metabolic processes (GO: 0051186) were also overrepresented, which either belongs to porphyrin (GO: 0006778) or glutathione (GO: 0006749) metabolic processes. Regarding cellular scenes of these versatile processes mostly found in the plastids (GO: 0009536), including the chloroplast (GO: 0009507), mitochondria (GO: 0005739) and cytoplasmic membrane-bounded vesicles (GO: 0016023). Ribosome biogenesis and translation are located in the ribosomes (GO: 0005840) or nuclear transcription factor complexes (GO: 0005667). Similarly to GL2 regulation in the cold, molecular functions such as glutathione transferase activity (GO: 0004364) and rRNA binding (GO: 0019843) were over-represented.

### Pathway analysis

**Figure 4:**
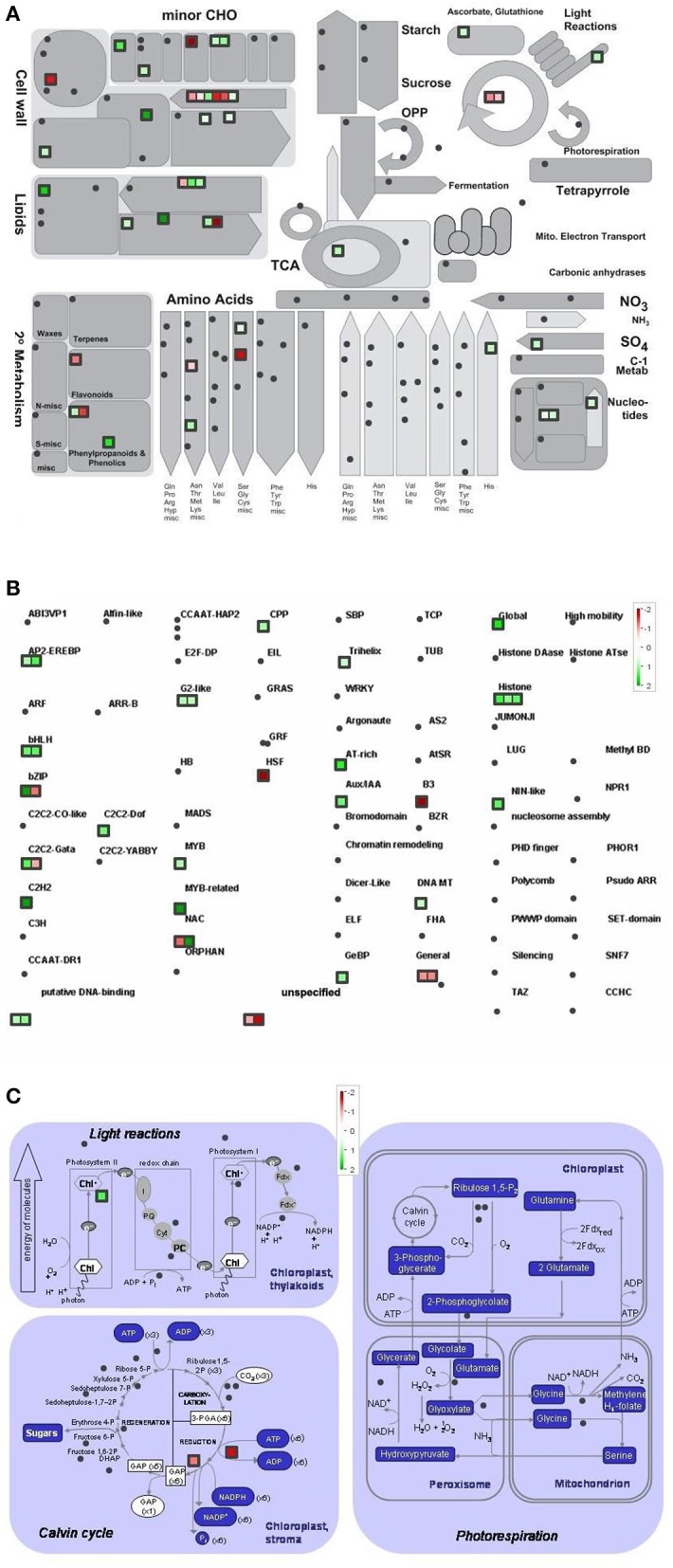
Metabolism overview **(A)** distribution of transcription factors **(B)** and genes involved in the photosynthesis **(C)** in Control vs. GL2 (effect of cold). logFC-values of the microarray-based expression were loaded into MapMan to generate the pathway map. Each square represents a logFC > |2| differentially expressed transcript. Genes whose expression does not change are coloured white, and an increasingly large increase or decrease is shown as an increasingly intense green or red colour, respectively. On the log2 scale ranging between 2 or 3 and −2 or −3, green and red colours represent 4, or 8-fold expression.

**Figure 5:**
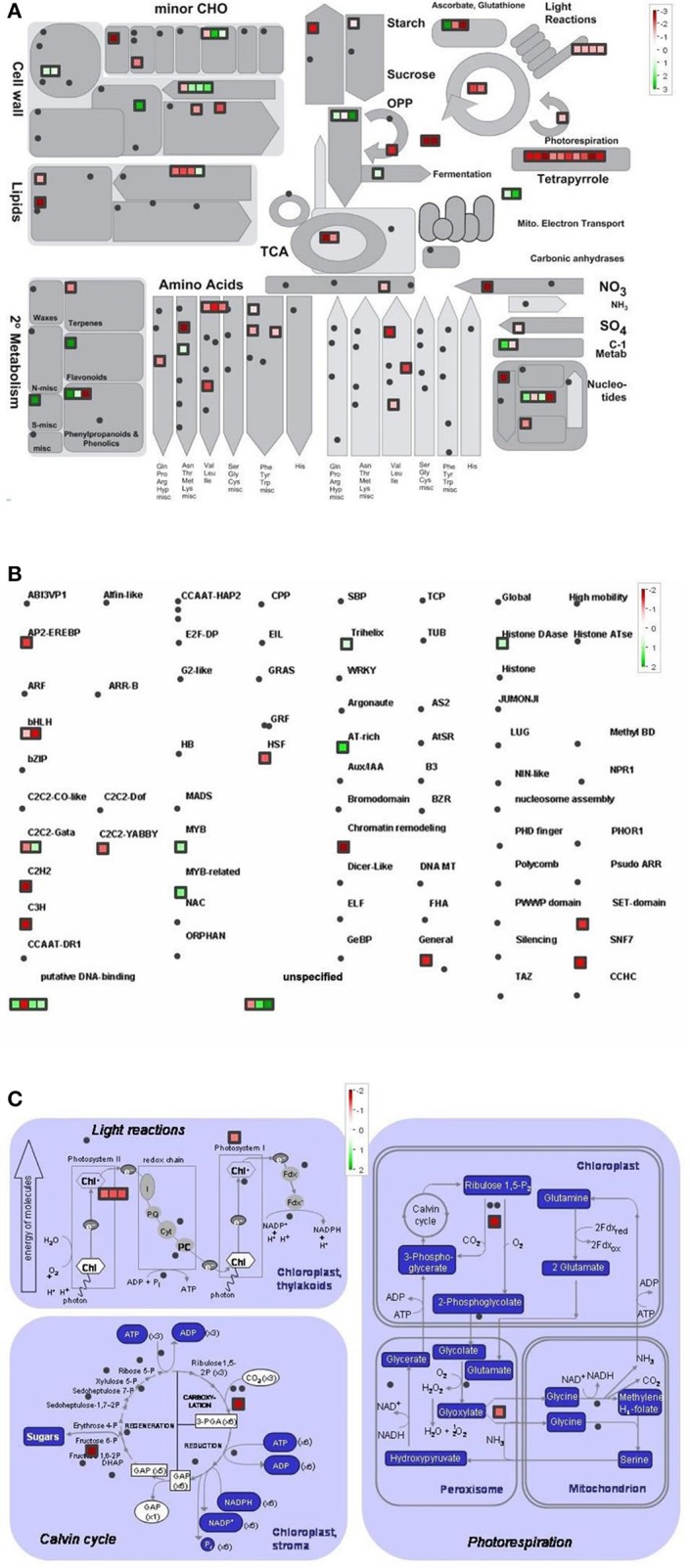
Metabolism overview **(A)** distribution of transcription factors **(B)** and genes involved in the photosynthesis **(C)** in GL2 vs. LL4 (effect of light). logFC values of the microarray-based expression were loaded into MapMan to generate the pathway map. Each square represents a logFC > |2| differentially expressed transcript. Genes whose expression does not change are coloured white, and an increasingly large increase or decrease is shown as an increasingly intense green or red colour, respectively. On the log2 scale ranging between 2 or 3 and −2 or −3, green and red colours represent 4, or 8-fold expression.

**Figure 6:**
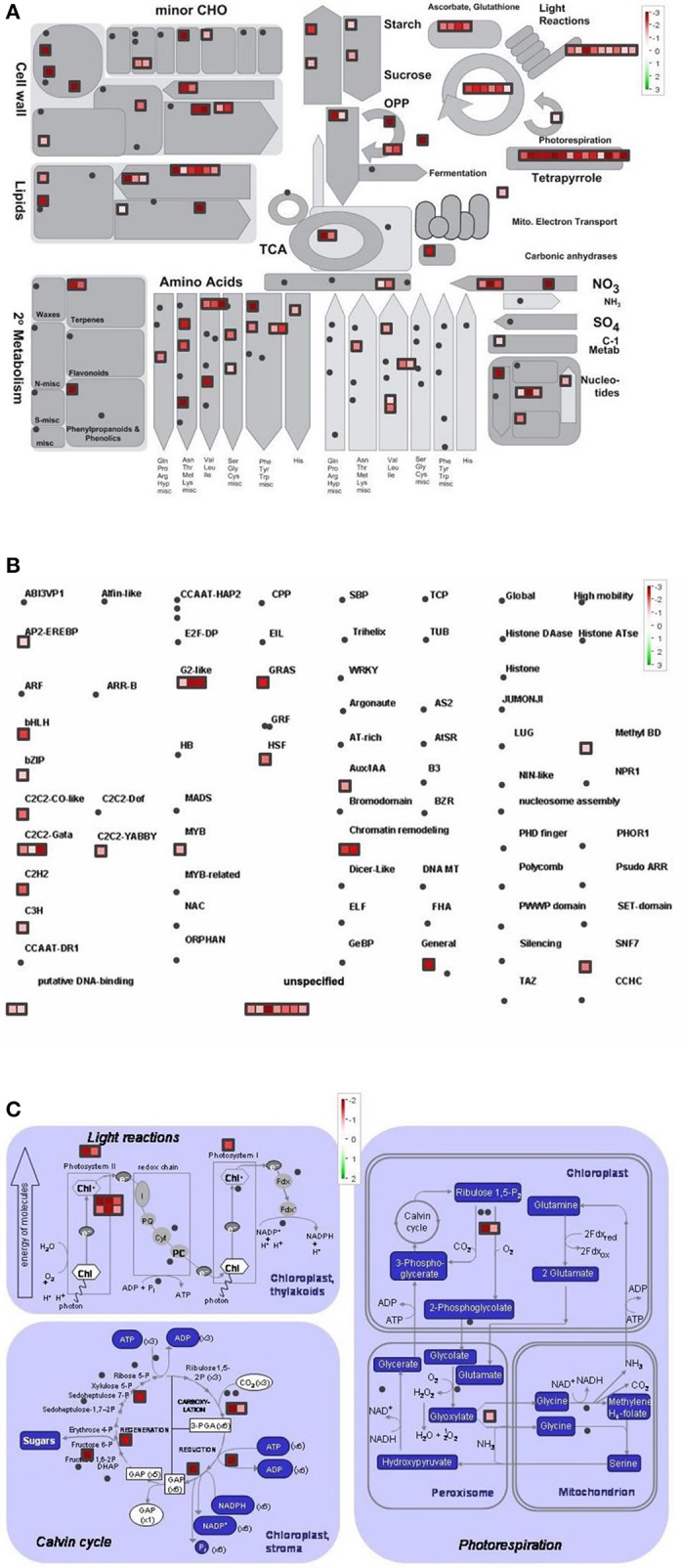
Metabolism overview **(A)** distribution of transcription factors **(B)** and genes involved in the photosynthesis **(C)** in Control vs. LL4 (effect of cold and light). logFC values of the microarray-based expression were loaded into MapMan to generate the pathway map. Each square represents a logFC > |2| differentially expressed transcript. Genes whose expression does not change are coloured white, and an increasingly large increase or decrease is shown as an increasingly intense green or red colour, respectively. On the log2 scale ranging between 2 or 3 and−2 or−3, green and red colours represent 4, or 8-fold expression.

Differentially regulated genes were classified into metabolic pathway groups using the MapMan software. Amongst numerous metabolic pathways, we found the most informative ones the followings: metabolic overview, transcription and photosynthesis (Figures 4–**6**).

The overview of cellular metabolism showed that both cold and light caused a remarkable rearrangement of metabolism. Generally, the effect of LL4 at low temperature caused the up-regulation of the genes, thus, facilitate the metabolism (Figures 4A, 5A, 6A). Both GL2 and LL4 influenced the tetrapyrrole metabolism in the cold. Similarly, the processes of light utilization on the PSII, carbohydrate metabolism and the ascorbate-glutathione cycle were induced rather at LL4 than at GL2. It can be concluded, that the most processes of assimilation are maintained by LL4 and repressed by cold or GL2.

The distribution of the expression of transcription factors is also affected by cold and light (Figures 4B, 5B, 6B). One of the most affected transcription protein family is the histones which were repressed at GL2 but not by LL4 in the cold. Both G proteins, bHLH, bZIPs and other putative DNA-binding proteins down-regulated by cold and GL2 and up-regulated by LL4. HSFs, interestingly, remained up-regulated in each treatments.

In a more detailed analysis, gene function of photosynthetic processes were dissected. Higher number of genes functions were classified into the photosynthetic processes at LL4 in the cold compared with control condition. Both light reactions and Calvin cycle were induced rather by LL4 repressed by GL2 (Figures 4C, 5C, 6C). Results suggest that the expression of genes related to photosynthetic processes is better influenced by light then by the temperature drop.

### Changes in the amount of soluble sugars

Some of the genes whose expression levels were significantly altered by the cold acclimating temperature and/or different light intensities during the hardening period, were involved in the carbohydrate metabolism (Table 1). The amounts of fructose, glucose, sucrose and maltose were determined in the leaves and roots after cold acclimation, after chilling treatment and during the recovery period. The highest fructose level was detected in the leaves of GL2 plants during hardening (Figure S15A) but dropped to the control level after three days at 5°C and remained at this level on the first day of recovery. Glucose and sucrose were detected in the highest amount in the leaves (Figures S15B,C). As in the case of fructose, a great increase was detected in the leaves of GL2 plants during hardening, but hardly any change was observed after 3 days at 5°C and only a slight decrease during recovery. A similar change was detected in the amount of maltose during hardening (Figure S15D), but its level increased at 5°C even in unhardened plants although the highest amount was measured in the leaves of GL2 plants. During the recovery period it dropped back to the initial level. Fructose and sucrose increased in the roots in a light-dependent manner during hardening (Figures S16A,C). The fructose content remained at the same level after 3 days at 5°C, but the amount of sucrose increased, particularly in the case of GL2 and LL3. No substantial changes were determined in the levels of glucose and maltose in the roots (Figures S16B,D).

**Table 1:** List of gene functions related to carbohydrate metabolism and regulated by cold, light or both factors.

**Cold-regulated**				**Light-regulated**				**Combined regulation**			
**Accession number**	**Gene function**	**log2FC**	***p-*****value**	**Accession number**	**Gene function**	**log2FC**	***p*****-value**	**Accession number**	**Gene function**	**log2FC**	***p*****-value**
NP_001130108	Glycerophosphodiester phosphodiesterase	3.9370346	0.002137337	NP_001151917	Phosphoenolpyruvate carboxylase kinase 1	4.1780014	5.85E-04	AQK60148	Glucomannan 4-beta-mannosyltransferase 2	4.190055	5.95E-05
AQK97088	Phosphoglycerate Mutase family protein	2.324909	0.002300885	ACG34953	Glycerol-3-phosphate Acyltransferase 8	2.4213006	0.003560609	ONM36349	Glycerol-3-phosphosphate Acyltransferase1	2.5158112	0.003560609
ONM56633	Bifunctional UDP-glucose 4-epimerase and UDP-xylose 4-epimerase 1	1.657018	6.30E-04	ACG28932	Glycosyltransferase	2.375742	0.002720237	ONM02814	Glycerophosphodiester phosphodiesterase GDPDL3	2.1040068	4.56E-04
ONM60638	Alpha-galactosidase 3	1.4812617	0.002564861	NP_001105047	Aldehyde dehydrogenase 5	2.2098408	0.001409359	ONM10264	Plastidial pyruvate kinase 1 chloroplastic	1.807035	2.80E-04
AQK87666	Trehalose-6-phosphate synthase12	1.4775023	1.85E-04	ONM30490	Glycosyltransferase family 61 protein	2.2052014	0.001584862	NP_001148138	Glycerol 3-phosphate permease	1.7980256	0.003462655
ACG38167	Xyloglucan endotransglycosylase/hydrolase protein 8 precursor	1.2368568	2.24E-04	ONM10098	Aldehyde oxidase1	2.1058974	0.00453268	NP_001105608	Pyruvate dehydrogenase (lipoamide) kinase 2	1.6757569	5.62E-05
NP_001105608	Pyruvate dehydrogenase (lipoamide) kinase 2	1.1424388	5.62E-05	NP_001148092	Aldehyde dehydrogenase, dimeric NADP-preferring	2.0147645	3.71E-05	ONL96977	6-Phosphofructo-2-kinase/fructose-26-bisphosphatase	1.661703	2.06E-04
AQK88045	Beta-galactosidase 17	1.0441507	9.79E-04	ONM02814	Glycerophosphodiester phosphodiesterase GDPDL3	1.6459553	4.56E-04	NP_001137118	Aldose 1-epimerase precursor	1.4921634	0.00407572
NP_001338725	Ribulose bisphosphate carboxylase small subunit 2	−0.9178931	5.26E-04	ONM56302	Beta-glucosidase 11	1.2667857	4.08E-04	ONM22374	Glucose-6-phosphate isomerase 1 chloroplastic	1.3741354	0.00144248
XP_008675104	Alpha-amylase 3, chloroplastic	−0.9569126	1.05E-05	XP_008665592	Pyruvate kinase isozyme A, chloroplastic	1.1613737	2.80E-04	ONM62564	Aldehyde dehydrogenase	1.3029907	3.71E-05
ONL97046	Malonyl CoA-acyl carrier protein transacylase	−1.3121262	4.21E-05	ONL96977	6-phosphofructo-2-kinase/fructose-26-bisphosphatase	0.9451744	2.06E-04	AAN40021	Putative sugar transporter protein	1.286771	4.21E-06
AQK99444	Mannose-1-phosphate guanylyltransferase 1	−1.5589914	5.68E-06	AQK95232	Phosphoglycerate mutase-like protein AT74H	0.90894777	6.83E-04	ACG29374	Sugar carrier protein C	1.266195	0.002126372
XP_008656415	phosphoglycerate kinase, chloroplastic	−1.664048	1.74E-04	NP_001288421	Sugar transport protein 6-like	0.8480516	3.54E-04	AQK95232	Phosphoglycerate mutase-like protein AT74H	1.036925	6.83E-04
XP_008669175	probable inactive beta-glucosidase 14	−2.2985225	0.00177892	NP_001147854	6-Phosphofructokinase	0.7439168	5.28E-05	AAG22094	Ribulose 1,5-bisphosphate carboxylase/oxygenase activase precursor, partial	−0.99635535	2.49E-04
ONM00923	Alpha/beta-Hydrolases superfamily protein	−2.6030025	3.91E-04	ONM59878	4-Alpha-glucanotransferase DPE1 chloroplastic/amyloplastic	−0.80431604	4.06E-04	NP_001104840	Sucrose transporter 1	−1.0542811	0.004237142
				NP_001142302	Mannose-1-phosphate guanylyltransferase 1	−1.0449951	5.68E-06	ONM06244	Phosphoglycerate kinase	−1.1203326	6.00E-05
				NP_001151106	Fructose-1,6-bisphosphatase, cytosolic	−1.0704721	0.003318357	AQK69130	Sucrose-phosphate synthase 1	−1.3283775	3.44E-05
				ONM07834	Phosphoglycerate mutase-like family protein	−1.1184196	0.002953377	NP_001142404	Phosphoglycerate kinase	−1.4211287	0.003085656
				XP_008654210	trehalose-6-phosphate synthase isoform X1	−1.2203356	1.85E-04	ONM00332	Glycerol-3-phosphate acyltransferase chloroplastic	−1.4539771	3.02E-04
				ONM00332	Glycerol-3-phosphate acyltransferase chloroplastic	−1.25687	3.02E-04	AQL07810	Alpha/beta-Hydrolases superfamily protein, partial	−1.6154816	0.002821473
				XP_008645148	phosphoglycerate kinase isoform X1	−1.2609754	6.00E-05	XP_008673647	Phosphoenolpyruvate/phosphate translocator 3, chloroplastic	−1.6809244	5.76E-05
				AAG22094	Ribulose bisphosphate carboxylase/oxygenase activase, chloroplastic precursor	−1.3490247	0.002121331	AAX07938	Phosphoenolpyruvate carboxylase kinase 4	−1.8123076	5.22E-04
				AQK62551	Ribose-5-phosphate isomerase	−1.3925734	0.001351543	XP_008656415	phosphoglycerate kinase, chloroplastic	−2.149561	1.74E-04
				XP_020403676	Ribulose bisphosphate carboxylase/oxygenase activase 2, chloroplastic	−1.4514501	0.001175892	XP_020403676	Ribulose bisphosphate carboxylase/oxygenase activase 2, chloroplastic	−2.1777864	0.001175892
				NP_001130532	Beta-galactosidase Precursor	−1.5497097	3.79E-04	ACF87565	Unknown	−2.2316277	0.001227283
				AQL09362	Sucrose transport protein SUC3	−1.5682054	1.30E-04	NP_001147459	Fructose-1,6-bisphosphatase	−2.2366343	0.002749594
				AQK85634	Mannosylglycoprotein endo-beta-mannosidase	−1.6499152	0.003568029	NP_001108121	Starch branching enzyme III	−2.3248887	4.09E-04
				AQK55931	Trehalose-6-phosphate synthase10	−1.6655805	8.54E-04	AQK99444	Mannose-1-phosphate guanylyltransferase 1	−2.6039865	5.68E-06
				AAX07938	Phosphoenolpyruvate carboxylase kinase 4	−1.7103847	5.22E-04	ONM18906	Pullulanase-type starch debranching enzyme1	−2.8656135	1.51E-05
				XP_020401744	ruBisCO large subunit-binding protein subunit alpha, chloroplastic	−1.799408	2.64E-04	ONL97046	Malonyl CoA-acyl carrier protein transacylase	−3.1654654	4.21E-05
				ACG30583	Trehalose-6-phosphate synthase	−1.8578088	5.82E-04	AAC49012	Xyloglucan endo-transglycosylase homolog; similar to Triticum aestivum endo-xyloglucan transferase, PIR Accession Number E49539	−3.4923546	8.17E-04
				NP_001108121	Starch branching enzyme III	−1.9227829	4.09E-04	ONM00923	Alpha/beta-Hydrolases superfamily protein	−3.842009	3.91E-04
				XP_008659412	Alpha-galactosidase 3	−1.9270322	3.29E-05				
				XP_008659412	Alpha-mannosidase	−1.9270322	3.29E-05				
				ONM34741	Alpha/beta-Hydrolases superfamily protein	−1.9846559	2.95E-05				
				NP_001147459	Fructose-1,6-bisphosphatase	−2.1469374	0.002749594				
				ONM29674	Phosphoenolpyruvate/phosphate translocator 3, chloroplastic	−2.2858033	5.76E-05				
				AQK65349	Starch synthase IIc	−2.3964448	0.001227283				
				NP_001104914">	Pyruvate dehydrogenase 2	−3.0675209	4.73E-06				
				NP_001105775	Alkaline alpha galactosidase 3	−3.1119506	4.45E-05				

*Lists were ordered according to the log2FC values (accession number and gene function derived from a BLASTX search in nr database in maize, log2FC and p-value derived from the array image processing)*.

## Discussion

Exposure to low, but non-freezing temperatures is necessary for the development of the maximum level of freezing tolerance in winter cereals, even in the case of the most hardy genotypes, but it has long been known that the development of freezing tolerance is inefficient at low light intensity (Gray et al., 1997). Since it is possible for even chilling-sensitive maize plants to become acclimated to relatively low temperatures (Anderson et al., 1994; Janda et al., 1998), an analogous experiment was carried out to elucidate how light contributes to the development of cold acclimation processes in maize plants. In the first experiment, maize plants were grown at relatively low light intensity (GL1), which was further reduced during the acclimation period. Measurements of electrolyte leakage from the leaves suggested that, similarly to the frost hardening of winter cereals (Gray et al., 1997; Apostol et al., 2006), cold acclimation was less efficient at low light intensity in maize plants. The MDA level in the leaves after exposure to low temperature (5°C) was also higher in plants cold acclimated at low light intensity than at GL1. Interestingly, the chlorophyll content in the leaves showed an opposite pattern to the MDA content in the roots.

These results indicated that although light is necessary during the acclimation period to achieve improved chilling tolerance, it may also have various “side effects.” Photoinhibition often occurs under stress conditions, when the photosynthetic processes are impaired or negatively affected by unfavourable environmental factors (Long et al., 1983). Low temperature-induced photoinhibition has been widely studied in a wide range of chilling-sensitive plant species (Janda et al., 1994; Gómez et al., 2015). It has also been shown that upon exposure to low temperature in the light not only photosynthetic processes, but also other stress-related mechanisms are affected, such as the polyamine or free amino acid metabolisms (Szalai et al., 1997). To reveal further details of the effect of light during the cold acclimation period, another experiment was designed with higher light intensities. It has been demonstrated in maize plants that several stress symptoms cannot be detected at low temperature. They only appear in the post-chilling period (Szalai et al., 1996). Fluorescence induction measurements indicated that hardening at higher light intensities reduced the quantum efficiency of Photosystem 2 at low temperatures and also led to the slower recovery of this parameter at normal growth temperature. Interestingly, after exposure to low temperature, plants acclimated at higher light intensities had higher Y(NO), which reflects the fraction of energy that is passively dissipated, and lower Y(NPQ), which represents the regulated non-photochemical quenching, than plants cold acclimated at lower light intensity or not hardened at all. Since the occurrence of high Y(NO) in the light-adapted state is usually thought to indicate the presence of severe stress (Lazár, 2015; Zhang et al., 2017), these results suggest that cold acclimation at high light intensity also stresses plants. However, visual analysis clearly demonstrated that, in spite of the occurrence of photoinhibition, light (GL2) was still beneficial in terms of cold tolerance. These results suggest that the fact that photoinhibition is a possible source of reactive oxygen species may also have useful effects, such as inducing stress acclimation processes in plants (Foyer et al., 2017). Although increasing light intensity itself may also induce cold tolerance to a certain degree, as demonstrated for cereals (Janda et al., 2014), and although it has been shown that the excitation pressure in the photosynthetic electron transport chain is a critical component of the induction of cold tolerance, it cannot be stated that a high level of cold tolerance can be achieved simply through photoinhibitory treatment. It occurs under various type of stress conditions, such as drought, heat, etc. (whenever carbon fixation is limited), but the plants do not necessarily become cold-tolerant (although cross-tolerance effects have been reported in some cases). Nevertheless, it seems that photoinhibition may function as a signal not only for the development of cold hardiness in cereals but also, as suggested by the present results, for the development of cold acclimation in maize.

In efforts to understand the genetic basis of cold sensitivity in maize, several genes responding to low temperature have been identified. The recently published results of microarray-based genomic analysis indicated that hundreds of genes were affected by chilling treatment of various durations (Trzcinska-Danielewicz et al., 2009). Earlier studies showed changes in the expression of various specific genes in maize plants exposed to low-temperature stress. Cold-regulated genes have several functions, influencing transcription factors and genes related to DNA methylation, carbohydrate and secondary metabolism, etc. (Marocco et al., 2005). Among the transcripts present at higher levels in maize seedlings after cold acclimation were those corresponding to chilling acclimation-responsive genes encoding maize mitochondrial catalase isozymes (Anderson et al., 1994). In the cold tolerant Arabidopsis plants it has been shown that the levels an Arabidopsis bZIP transcription factor HY5, which has a pivotal role in light signalling are also regulated by low temperature transcriptionally, via a C-repeat binding factor- and abscisic acid-independent pathway, and post-translationally, via protein stabilization through nuclear depletion of a crucial repressor of light signalling CONSTITUTIVE PHOTOMORPHOGENIC 1 (COP1). HY5 also positively regulated the cold-induced gene expression through certain cis-acting elements, ensuring the complete development of cold acclimation (Catalá et al., 2011).

Recent analyses on the chilling sensitive maize plants indicated three main mechanisms that could be responsible for cold tolerance: the modification of the photosynthetic apparatus, cell wall properties and developmental processes (Sobkowiak et al., 2016). The regulation of the amount of reactive oxygen species and of redox homeostasis have been demonstrated to be important for cold acclimation processes in various plant species (Fracheboud et al., 2004; Gill and Tuteja, 2010; Kocsy, 2015; Boldizsár et al., 2016a). In the present work the comparison of the gene expression profiles in control and acclimated plants demonstrated that the light intensity is at least as important factor as the temperature during the cold acclimation period. The analysis of gene classes with a significant response to the treatments revealed complex regulation mechanisms and interactions between cold and light signalling processes. These results highlight the significance of numerous significantly differentially expressed genes, which are involved in most of assimilation and metabolic pathways, namely photosynthetic light capture via the modification of chlorophyll biosynthesis and the dark reactions, carboxylic acid metabolism, cellular amino acid metabolic processes, such as serine, threonine and glutamine metabolism, cofactor, porphyrin or glutathione metabolic processes as well as ribosome biogenesis and translation. It suggests that light intensity has a fundamental influence on the whole assimilation and metabolism at suboptimal temperature in maize.

In the present study detailed analysis was carried out focusing on the separate effects of cold and light, with special regard to Control vs. GL2 and GL2 vs. LL4 and Control vs. LL4 comparisons. The pathway analysis of gene functions suggests that both light reactions and Calvin cycle were induced by LL4 and repressed by GL2 at low temperature. It is known, that temperature, including chilling and heat, and light prominently influence chloroplast development and chlorophyll biosynthesis, which affect photosynthesis efficiency. Most enzymes involved in the tetrapyrrole pathway significantly differentially expressed in the present experiment. Our results show that the chlorophyll biosynthetic processes were repressed both by the low temperature and GL2. At low light intensity (LL4), however, these genes were up-regulated. In accordance to our findings, chlorophyll biosynthesis was also found to be greatly light-dependent in wheat and cucumber due to a lower activation some key enzymes of tetrapyrrole biosynthesis in the light at suboptimal temperatures (Mohanty et al., 2006).

Photosynthesis is in a strong relation with amino acid biosynthesis. Chloroplasts are able to reduce nitrite to ammonia, and synthesize a number of amino acids (Wallsgrove et al., 1983). Not surprisingly, amino acid metabolism is also promoted by low light and not by normal growth light in the cold. The pathway analysis of the gene functions classified into transcription factor families showed that more genes were silenced by GL2 than LL4. This can be related to the higher number of active assimilation processes acting under low light during the cold period.

In contrast to the amino acid metabolism, besides many pivotal and secondary biological processes, the translation and ribosome biogenesis, the most important influence of cold was found on the cellular carbohydrate metabolism, particularly hexose biosynthetic process. Beside other processes, GL2 had also significant effect on the hexose biosynthesis in the cold. Literature data also show, that cold acclimation is often associated with the accumulation of compatible solutes (sugars, proline, etc.) (Thomashow, 1999). The present work also focused on the involvement of changes in soluble sugars in osmotic regulation. Soluble sugars may function as osmoprotectants, buffering the harmful effect of low temperatures. The accumulation of soluble sugars was found to be correlated with enhanced freezing tolerance in *Arabidopsis* leaves (Wanner and Junttila, 1999). An elevated level of sugars was also detected during cold acclimation in tomato plants (Song et al., 2015). The shoots of a freezing-tolerant pea genotype accumulated three times more sucrose than the shoots of a freezing-sensitive genotype at low but non-freezing temperature (Grimaud et al., 2013), suggesting that the exposure of tolerant pea plants to low temperatures activates the gluconeogenesis pathway, leading to the accumulation of soluble sugars. In a similar manner, the highest soluble sugar accumulation during the hardening of plants was detected at relatively high light intensity in the present work. This rise in sugar content may also contribute to the better chilling tolerance of maize plants. Chilling at night also caused a significant increase in the soluble sugar content in *Hevea brasiliensis* (Tian et al., 2016). The present results showed that while acclimation at moderately low temperature (15°C) and low light intensity reduced the level of glucose and sucrose, chilling at 5°C increased the levels of soluble sugars. At gene expression level, the effect of light was more emphasized than the cold itself. In both cases, greater number of down-regulated genes involved in the carbohydrate metabolism were found. There was no overlap found between carbohydrate-related genes induced either by cold or light. On the other hand, the gene expression of alpha/beta-hydrolases and the alpha-galactosidase 3 was silenced both at low temperature and under LL4. Similarly, the alpha-galactosidase proteins from barley and *Arabidopsis* were described with an important during dark induced senescence and has roles in leaf development by functioning in cell wall loosening and cell wall expansion (Chrost et al., 2007). The alpha/beta hydrolase superfamily proteins have versatile interactions of G-protein signalling under stress conditions (Khatri and Mudgil, 2015).

## Conclusions

The light intensity during the cold acclimation period may significantly affect cold acclimation processes in chilling-sensitive maize plants. Although photoinhibition may be dominant even at relatively high light intensity, light nevertheless contributes to the development of cold acclimation in maize. Interestingly, certain stress responses are light-dependent not only in the leaves, which sense the light directly, but also in the roots. As found earlier in frost-tolerant plants, light influences various light-related cold acclimation processes not directly, but at the gene expression and metabolomics levels. The results suggest that the photoinhibition induced by low temperature can be a necessary evil for cold acclimation processes in plants. The avoidance of exposure to high light intensity, especially at low temperature would appear to be required to protect the photosynthetic apparatus, but if the whole plant is to be protected from a subsequent cold shock, it may be necessary to accept the consequences of photodamage in the interests of acquiring cold-acclimated plants.

## Author contributions

GS designed the experiment, measured the sugars, and supervised the work. IM measured the chlorophyll-a fluorescence and made the bioinformatic analyses. MP and OG measured the lipid peroxidation, the electrolyte leakage, and the chlorophyll content. SR and CO made the validation of microarray. IM and BK carried out the annotation of microarray. TJ, IM, RV, and GS wrote the article.

### Conflict of interest statement

The authors declare that the research was conducted in the absence of any commercial or financial relationships that could be construed as a potential conflict of interest.

## Abbreviations

FC: fold change
GL1: growth light 1 (180 μmol m^−2^ s^−1^)
GL2: growth light 2 (387 μmol m^−2^ s^−1^)
LL1: low light intensity 1 (46 μmol m^−2^ s^−1^)
LL2: low light intensity 2 (14 μmol m^−2^ s^−1^)
LL3: low light intensity 3 (283 μmol m^−2^ s^−1^)
LL4: low light intensity 4 (107 μmol m^−2^ s^−1^)
MDA: malondialdehyde
Y(II): quantum yield of Photosystem 2
Y(NO): non-regulated non-photochemical quenching
Y(NPQ): regulated non-photochemical quenching.
