# Supplementary material for: Janus-Faced Nature of Light in the Cold Acclimation Processes of Maize
Source: Front Plant Sci. 2018 Jun 19;9:850. doi: 10.3389/fpls.2018.00850 (PMC6018404; doi:10.3389/fpls.2018.00850)
Supplement: Supplementary file 4 [file Presentation_1.PDF]

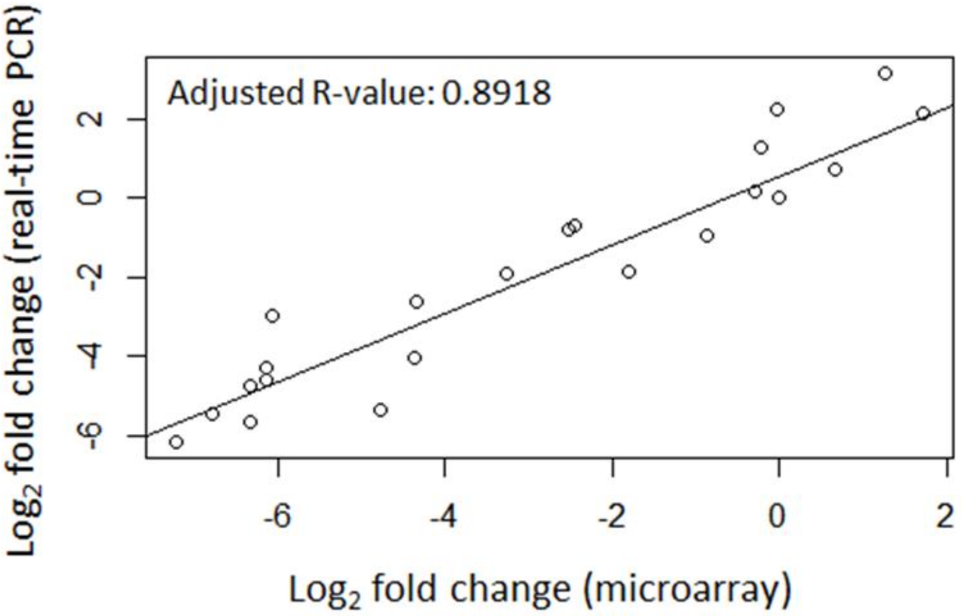

Figure S1. Validation of the microarray results. Microarray data (least-square means) were plotted against data from qRT-PCR and fitted into a linear regression. Both the x- and y-axes are shown on a log 2 scale.

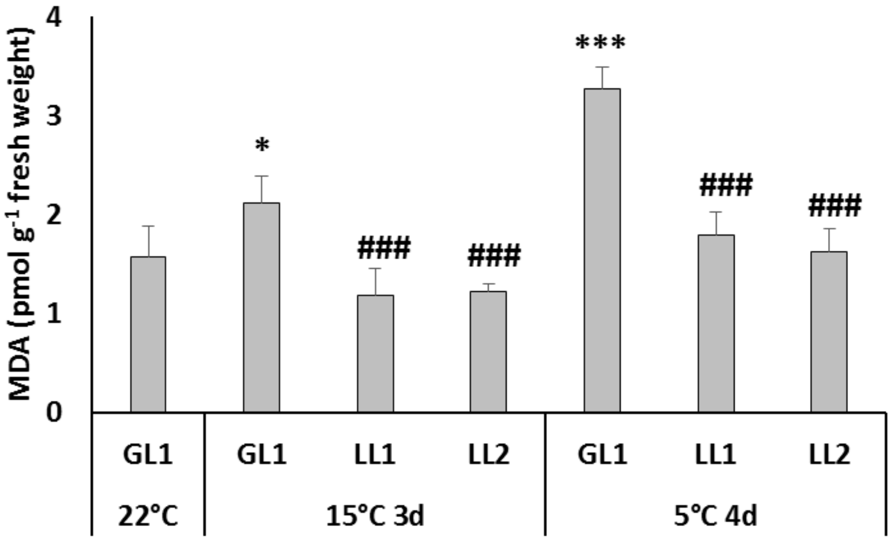

Figure S2. MDA level in the roots of plants grown at low light intensity before and after cold hardening at 15 °C, after chilling stress at 5 °C and recovery period. *,*** Significant differences compared to the control plants on the same day at the $p < 0.05$ and 0.001 levels, respectively. ### Significant difference compared to the GL2 plants on the same day at the $p < 0.001$ level (mean±SD, n=5).

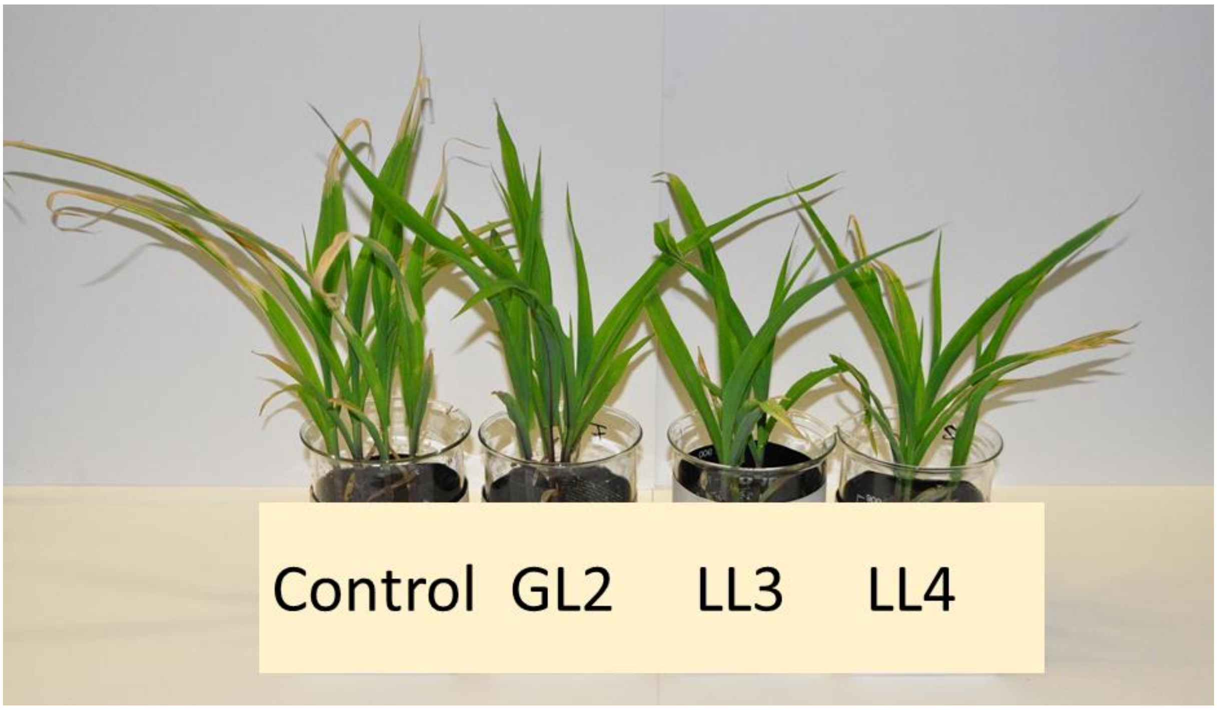

Figure S3. Representative plants after 3 days of chilling at 5°C followed by 4 days of recovery at 22 °C. From left to right: unhardened; hardened at GL2, hardened at LL3, and hardened at LL4.

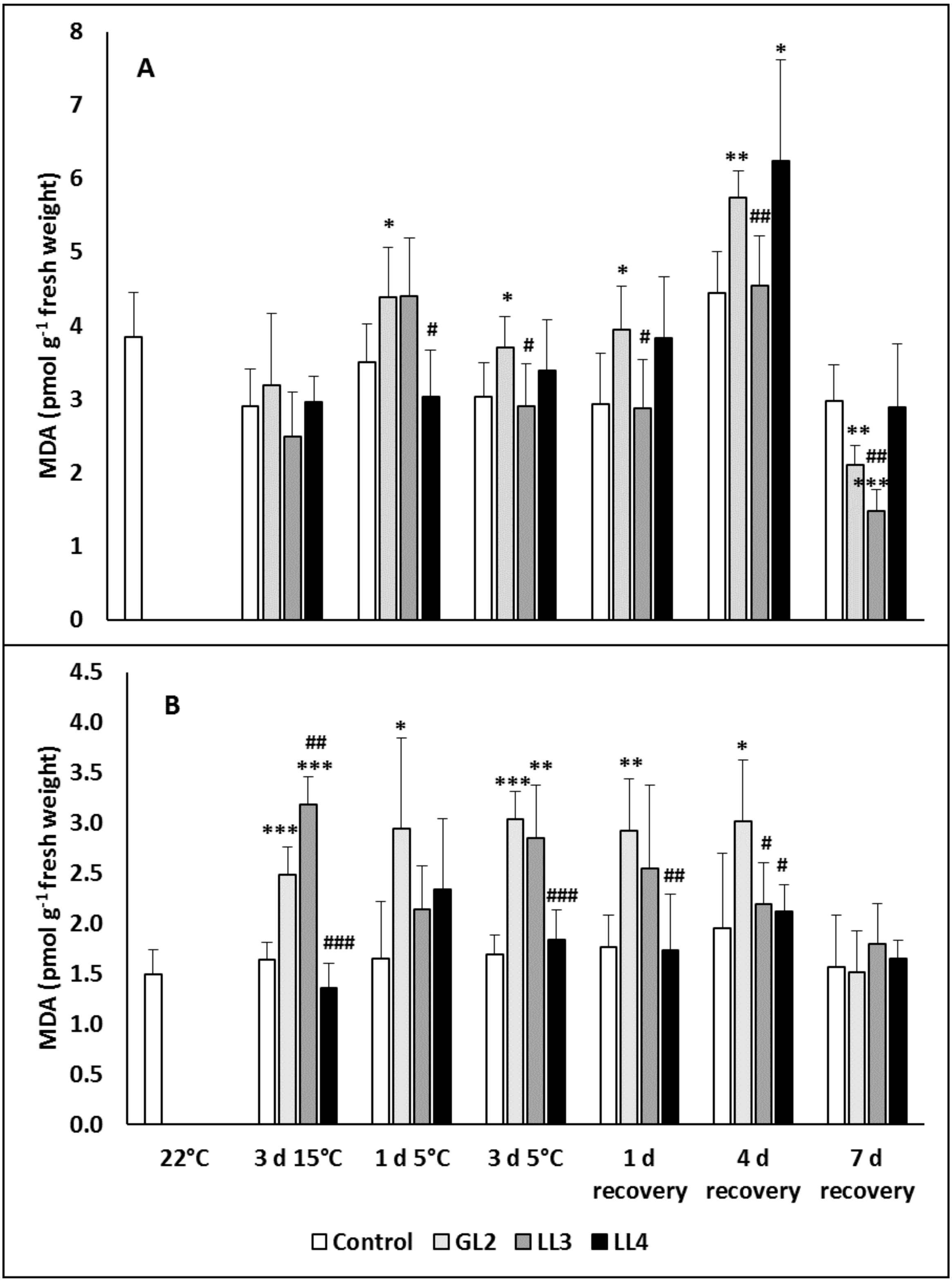

Figure S4. MDA levels in the leaves (A) and roots (B) of plants grown at low light intensity before and after cold hardening at 15 °C, after chilling stress at 5 °C and recovery period. *,,*** Significant differences compared to the control plants on the same day at the p < 0.05, 0.01 and 0.001 levels, respectively. #, ##, ### Significant differences compared to the GL2 plants on the same day at the p < 0.05, 0.01 and 0.001 levels, respectively (mean±SD, n=5).**

A: Up-regulated **B: Down-regulated**

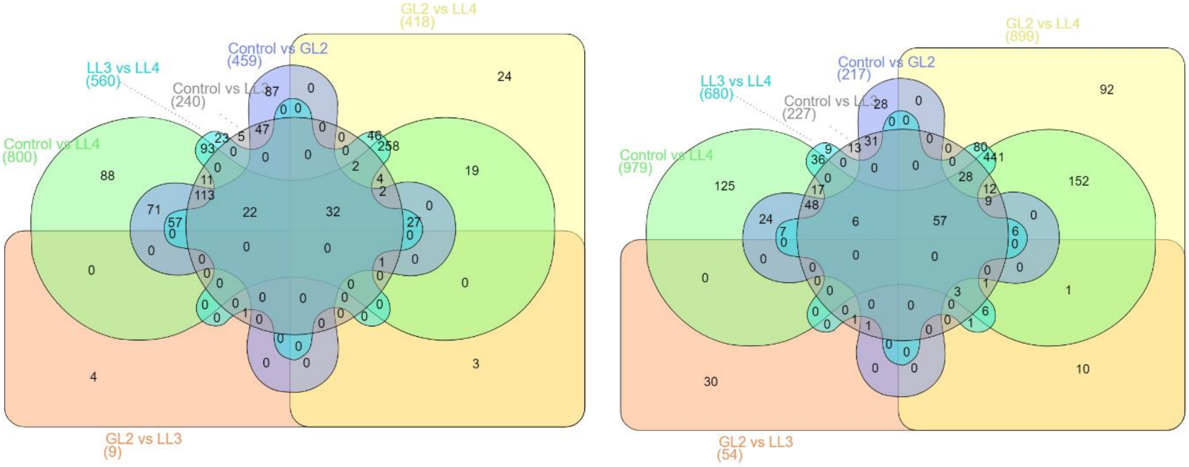

Figure S5. Venn diagrams show the number of up- (A) and downregulated (B) genes in the overlaps and those expressed individually after an $e<1^{-4}$ cutoff.

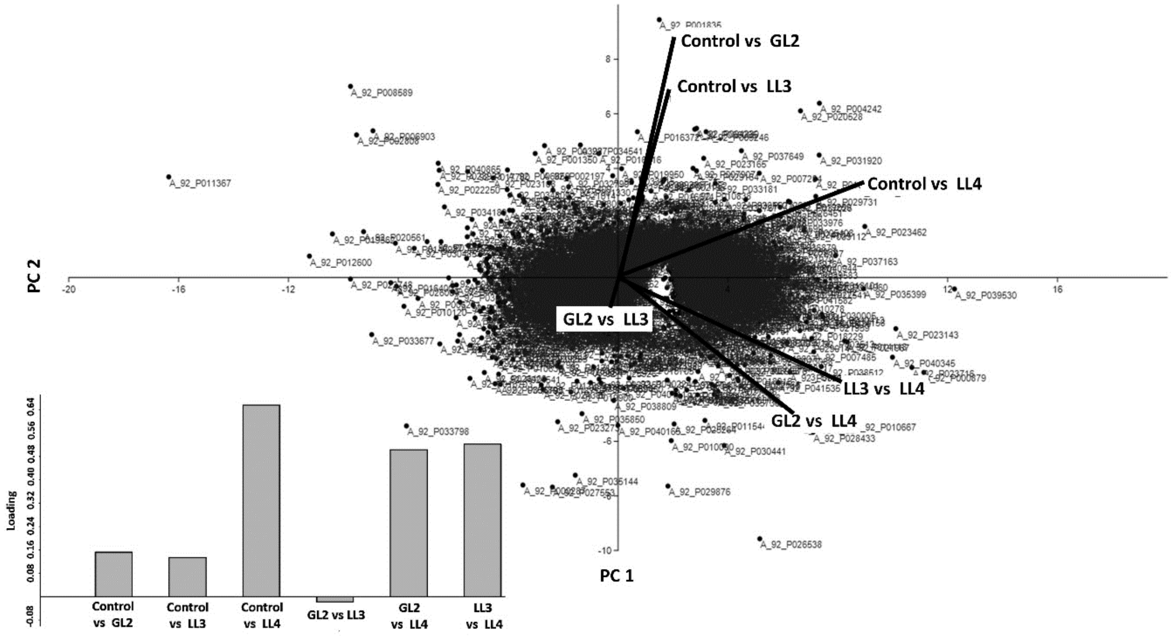

Figure S6. Scatter plot diagram of the expression datasets derived from principal component analysis based on the logFC values of the genes. The significant array probes were plotted with respect to the first and second principal components (PC1, PC2). The axes represent PC1 and PC2 and the biplot representation shows the projection of the original variables (light regimes in the cold) onto the scatter diagram. Insert: The loadings of the original variables along PC1.

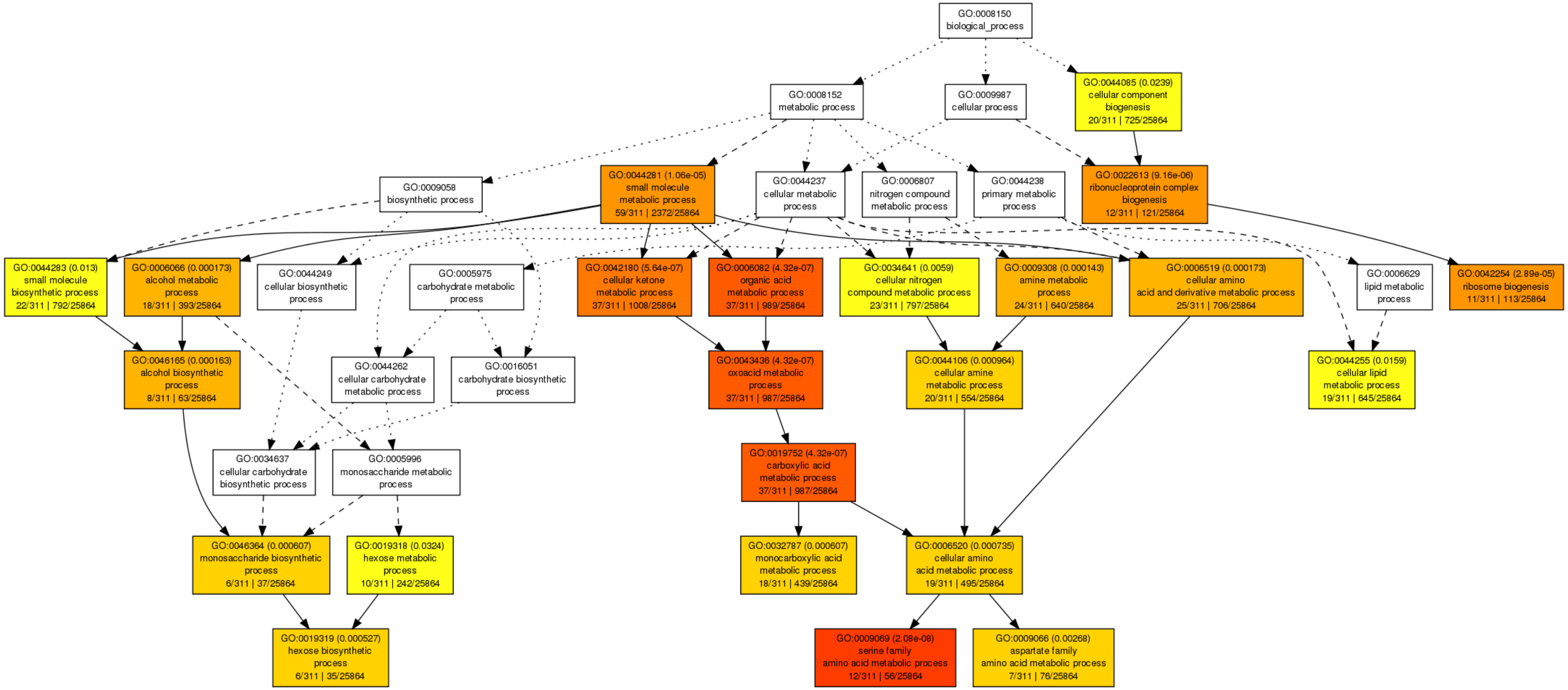

Figure S7. Gene ontology (GO) analysis carried out on the significantly differentially expressed microarray probes of Control vs GL2 comparison (cold-regulation) regarding to the GO class ‘biological process (BP)’. Overrepresented terms are shown in colored boxes (q < 0.05). Hierarchical graphs show the level of significance of the terms and the linkage among them. Box colors indicate levels of statistical significance: white = parent or non-significant term; yellow = 0.05, brown = 1e−5 and red = 1e-9. Arrow means conjunctions among terms: solid = is a; dashed = two significant nodes; dotted = one significant node, red = positive regulate and green = negative regulate. The pair of numerals in the left represents number of genes in query list associated with that GO term and number of genes in query list. The pair of numerals in the right represents number of genes associated with a particular GO term in the locus ID v3.30 (Gramene Release 50) database and total number of maize genes with GO annotations in the locus ID v3.30 (Gramene Release 50) database.

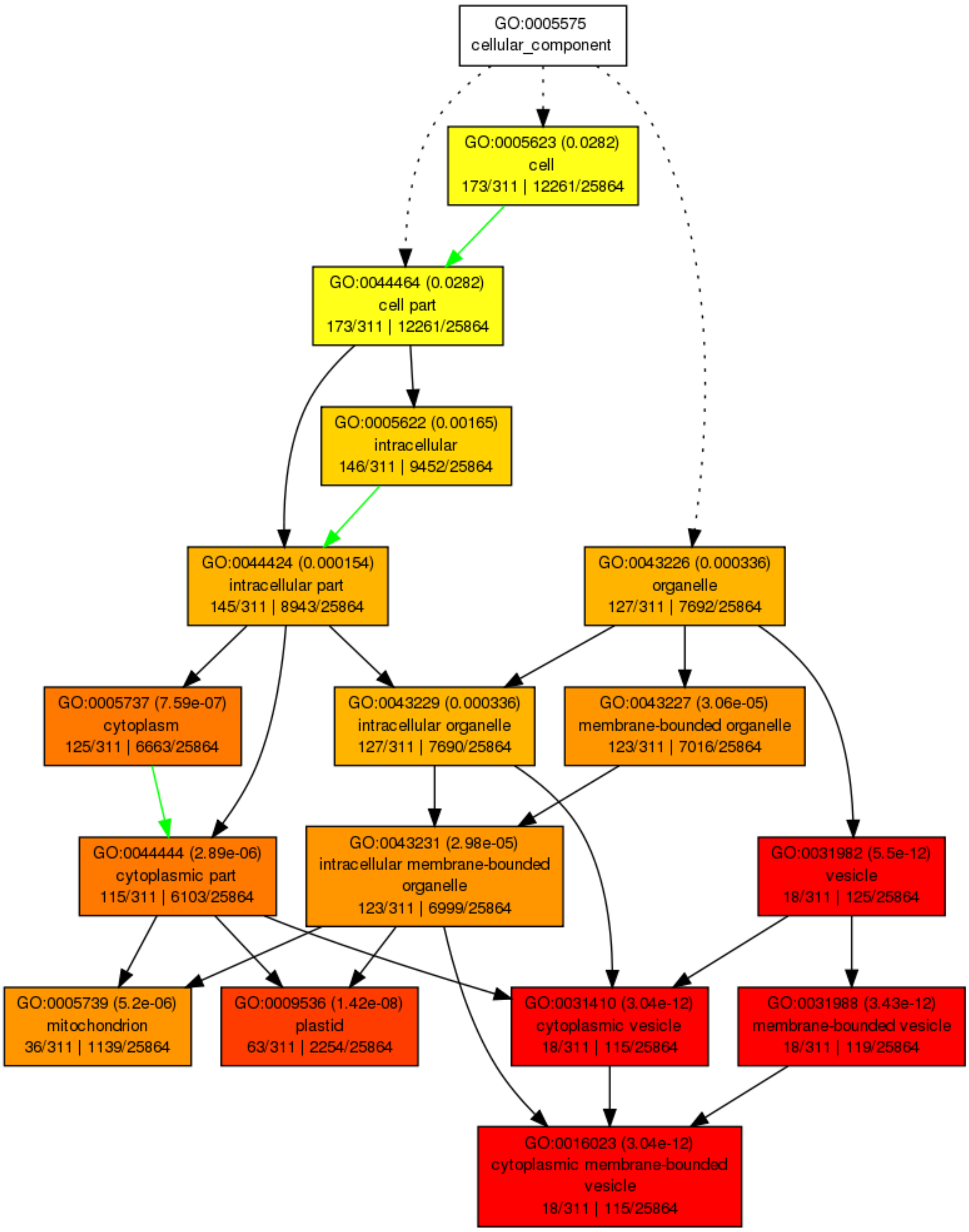

Figure S8. Gene ontology (GO) analysis carried out on the significantly differentially expressed microarray probes of Control vs GL2 comparison (cold-regulation) regarding to the GO class 'cellular component (CC)'. (MF)'. Overrepresented terms are shown in colored boxes (q < 0.05). Hierarchical graphs show the level of significance of the terms and the linkage among them. Box colors indicate levels of statistical significance: white = parent or non-significant term; yellow = 0.05, brown = 1e−5 and red = 1e-9. Arrow means conjunctions among terms: solid = is a; dashed = two significant nodes; dotted = one significant node, red = positive regulate and green = negative regulate. The pair of numerals in the left represents number of genes in query list associated with that GO term and number of genes in query list. The pair of numerals in the right represents number of genes associated with a particular GO term in the locus ID v3.30 (Gramene Release 50) database and total number of maize genes with GO annotations in the locus ID v3.30 (Gramene Release 50) database.

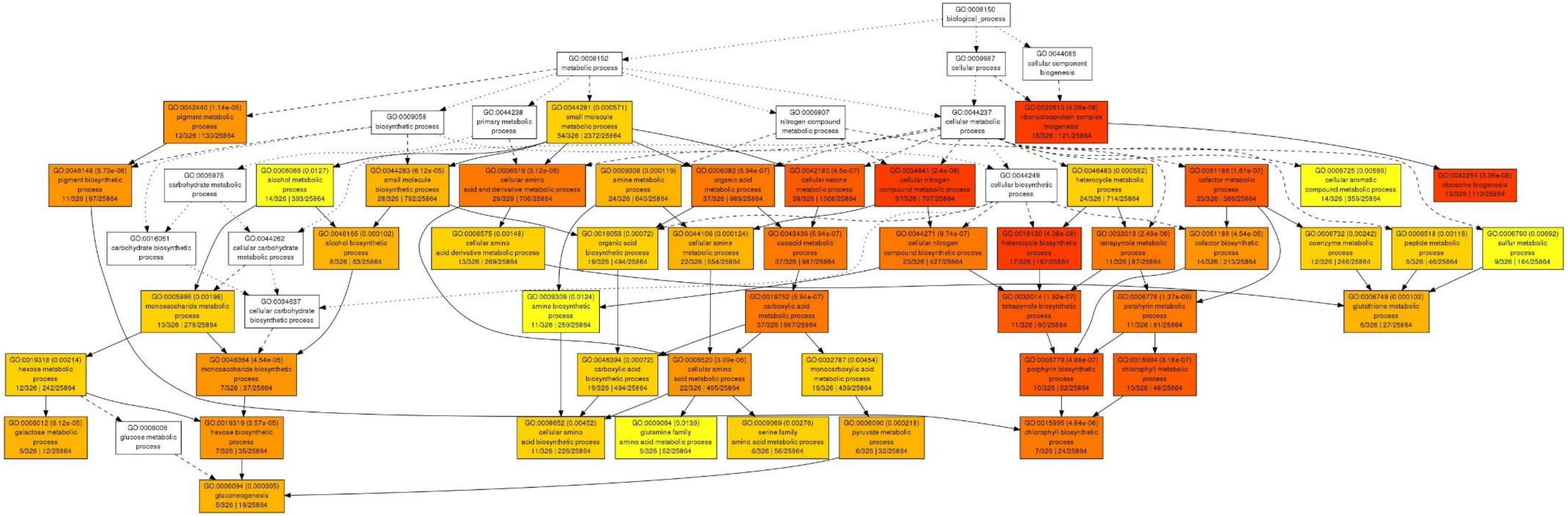

Figure S9. GO analysis carried out on the significantly differentially expressed microarray probes of GL2 vs LL4 comparison (light-regulation) regarding to the GO class 'biological process (BP)'. Overrepresented terms are shown in colored boxes (q < 0.05). Hierarchical graphs show the level of significance of the terms and the linkage among them. Box colors indicate levels of statistical significance: white = parent or non-significant term; yellow = 0.05, brown = 1e‒5 and red = 1e-9. Arrow means conjunctions among terms: solid = is a; dashed = two significant nodes; dotted = one significant node, red = positive regulate and green = negative regulate. The pair of numerals in the left represents number of genes in query list associated with that GO term and number of genes in query list. The pair of numerals in the right represents number of genes associated with a particular GO term in the locus ID v3.30 (Gramene Release 50) database and total number of maize genes with GO annotations in the locus ID v3.30 (Gramene Release 50) database.

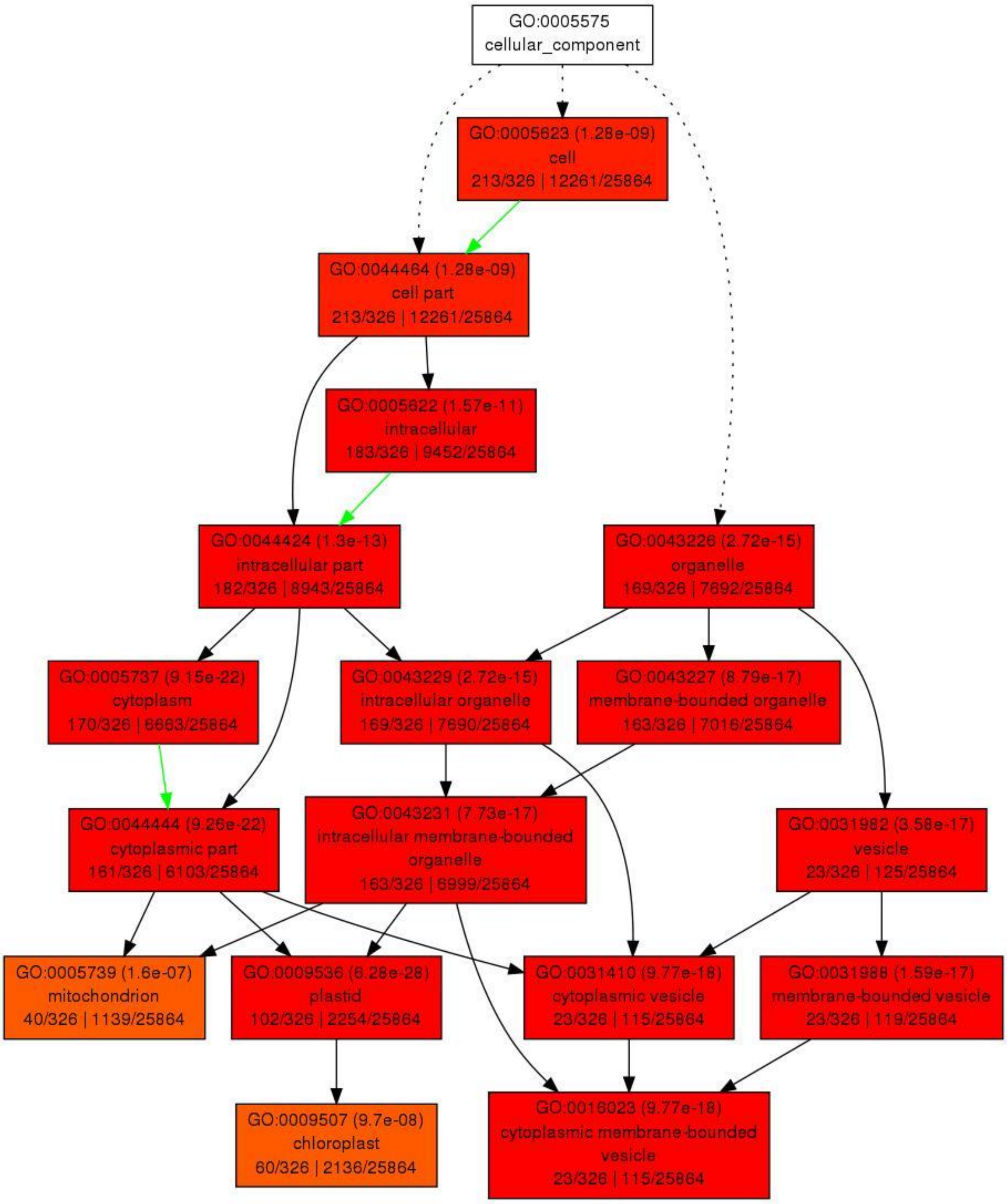

Figure S10. GO analysis carried out on the significantly differentially expressed microarray probes of GL2 vs LL4 comparison (light-regulation) regarding to the GO class 'cellular component (CC)'. Overrepresented terms are shown in colored boxes (q < 0.05). Hierarchical graphs show the level of significance of the terms and the linkage among them. Box colors indicate levels of statistical significance: white = parent or non-significant term; yellow = 0.05, brown = 1e−5 and red = 1e-9. Arrow means conjunctions among terms: solid = is a; dashed = two significant nodes; dotted = one significant node, red = positive regulate and green = negative regulate. The pair of numerals in the left represents number of genes in query list associated with that GO term and number of genes in query list. The pair of numerals in the right represents number of genes associated with a particular GO term in the locus ID v3.30 (Gramene Release 50) database and total number of maize genes with GO annotations in the locus ID v3.30 (Gramene Release 50) database.

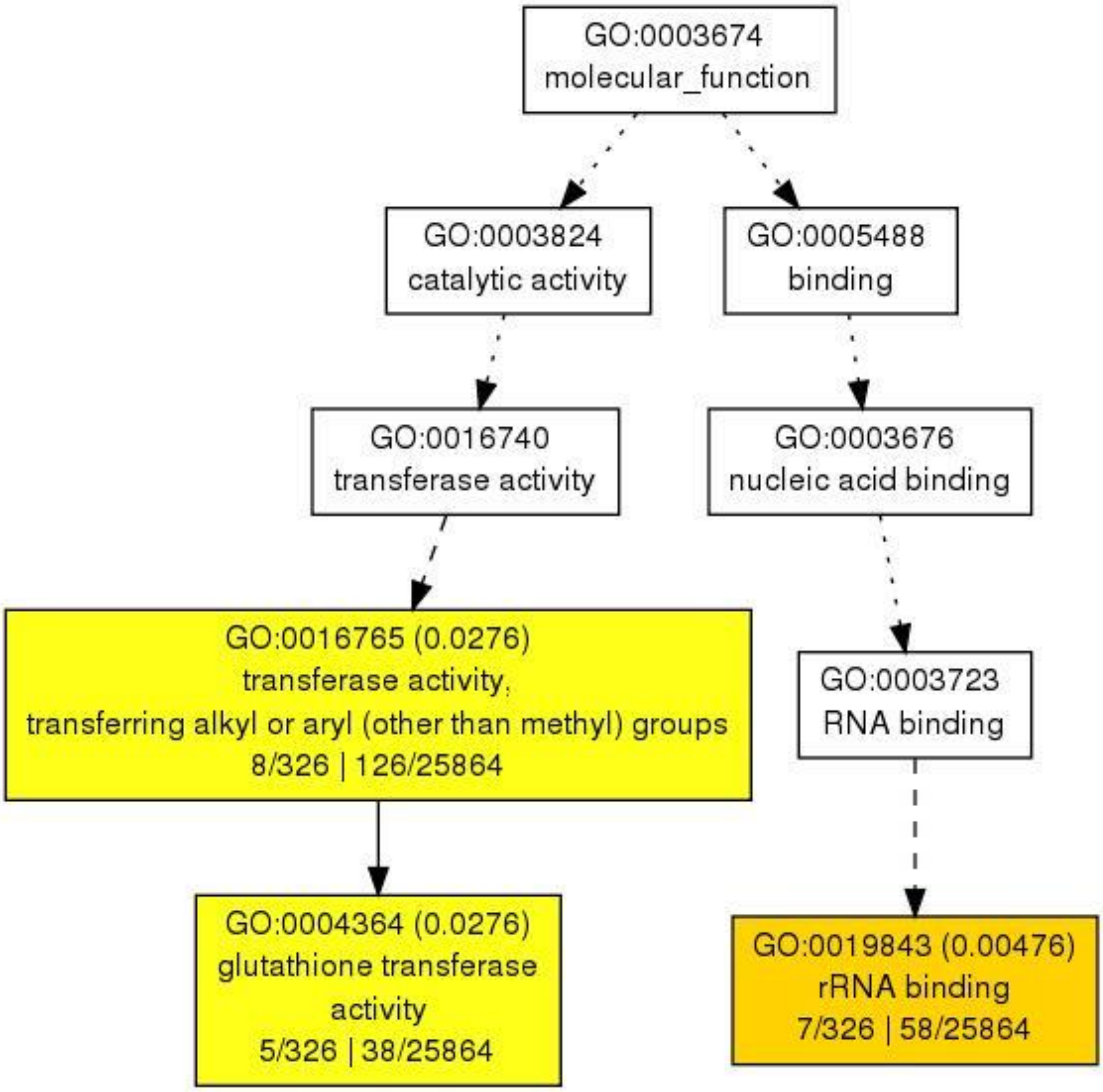

Figure S11. GO analysis carried out on the significantly differentially expressed microarray probes of GL2 vs LL4 comparison (light-regulation) regarding to the GO class 'molecular function (MF)'. Overrepresented terms are shown in colored boxes (q < 0.05). Hierarchical graphs show the level of significance of the terms and the linkage among them. Box colors indicate levels of statistical significance: white = parent or non-significant term; yellow = 0.05, brown = 1e−5 and red = 1e-9. Arrow means conjunctions among terms: solid = is a; dashed = two significant nodes; dotted = one significant node, red = positive regulate and green = negative regulate. The pair of numerals in the left represents number of genes in query list associated with that GO term and number of genes in query list. The pair of numerals in the right represents number of genes associated with a particular GO term in the locus ID v3.30 (Gramene Release 50) database and total number of maize genes with GO annotations in the locus ID v3.30 (Gramene Release 50) database.

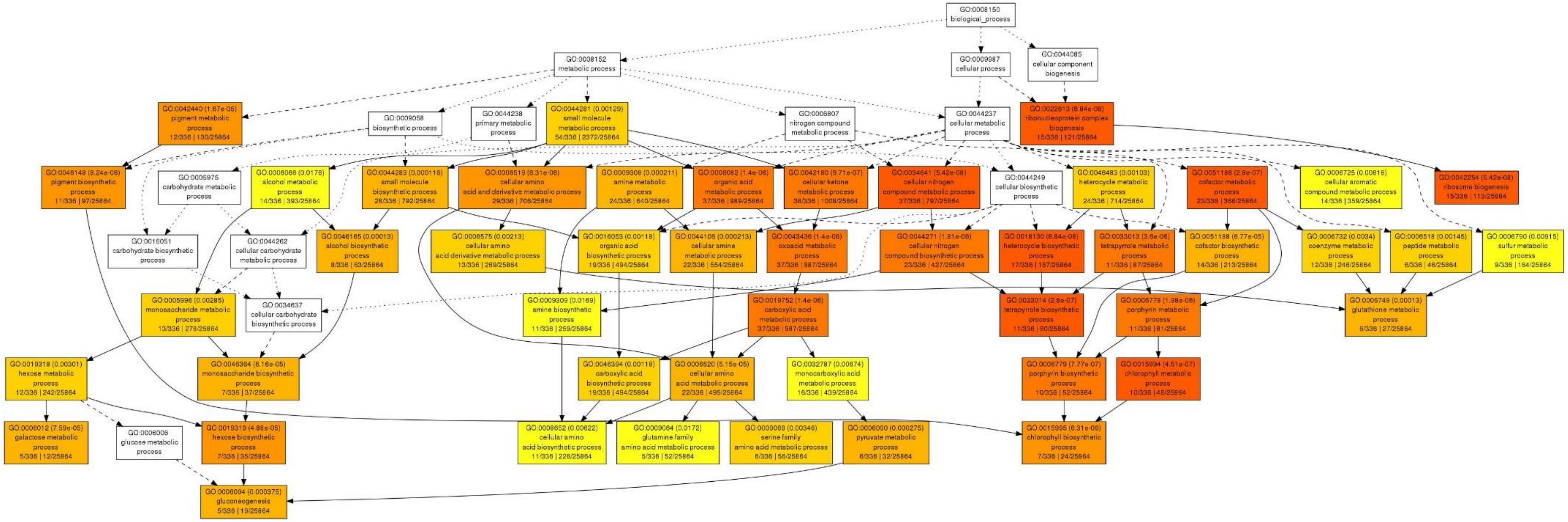

Figure S12. GO analysis carried out on the significantly differentially expressed microarray probes of Control vs LL4 comparison (cold- and light-regulation) regarding to the GO class 'biological process (BP)'. Overrepresented terms are shown in colored boxes (q < 0.05). Hierarchical graphs show the level of significance of the terms and the linkage among them. Box colors indicate levels of statistical significance: white = parent or non-significant term; yellow = 0.05, brown = 1e−5 and red = 1e-9. Arrow means conjunctions among terms: solid = is a; dashed = two significant nodes; dotted = one significant node, red = positive regulate and green = negative regulate. The pair of numerals in the left represents number of genes in query list associated with that GO term and number of genes in query list. The pair of numerals in the right represents number of genes associated with a particular GO term in the locus ID v3.30 (Gramene Release 50) database and total number of maize genes with GO annotations in the locus ID v3.30 (Gramene Release 50) database.

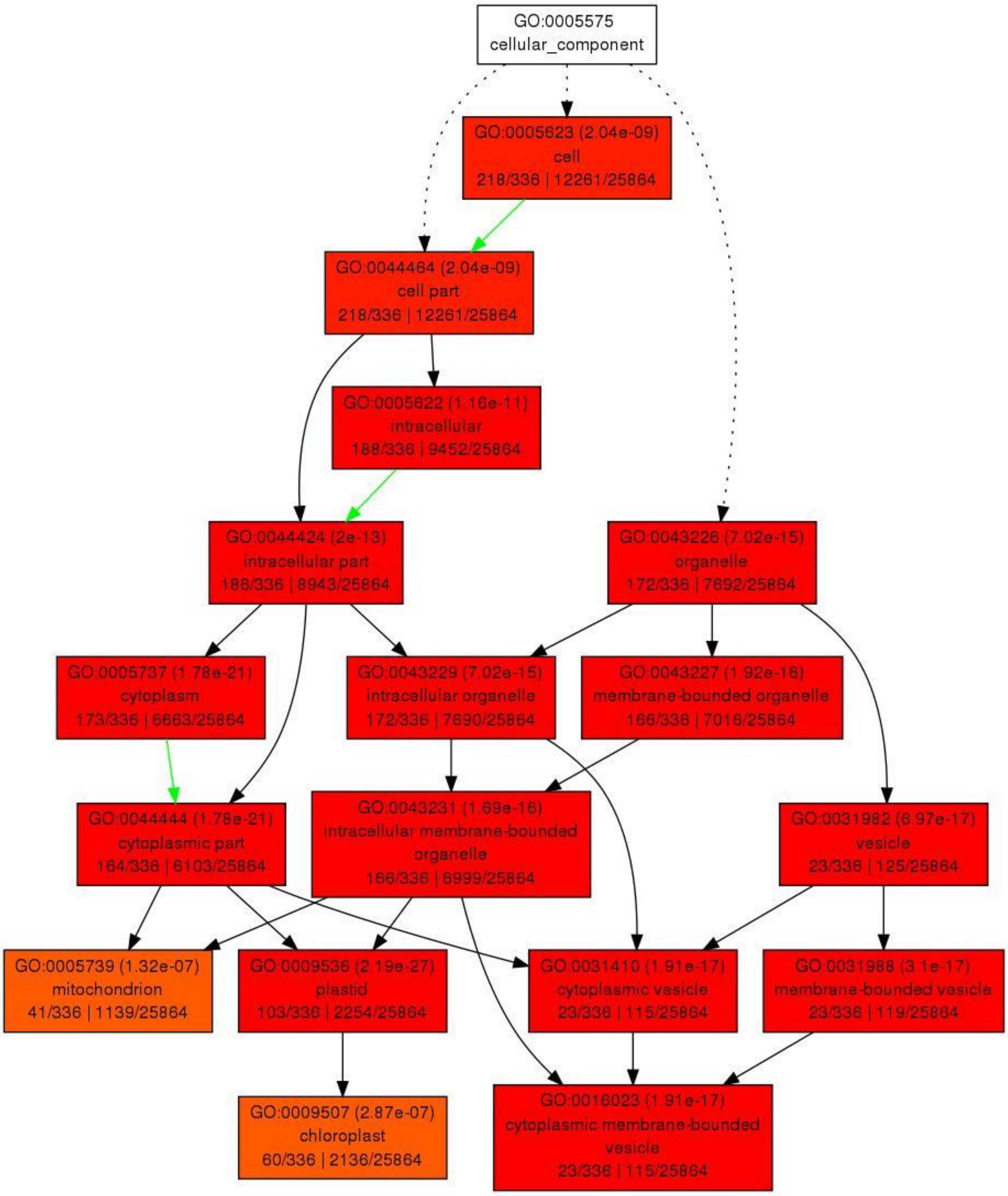

Figure S13. GO analysis carried out on the significantly differentially expressed microarray probes of Control vs LL4 comparison (cold-and light-regulation) regarding to the GO class 'cellular component (CC)'. Overrepresented terms are shown in colored boxes ($q < 0.05$). Hierarchical graphs show the level of significance of the terms and the linkage among them. Box colors indicate levels of statistical significance: white = parent or non-significant term; yellow = 0.05, brown = 1e−5 and red = 1e-9. Arrow means conjunctions among terms: solid = is a; dashed = two significant nodes; dotted = one significant node, red = positive regulate and green = negative regulate. The pair of numerals in the left represents number of genes in query list associated with that GO term and number of genes in query list. The pair of numerals in the right represents number of genes associated with a particular GO term in the locus ID v3.30 (Gramene Release 50) database and total number of maize genes with GO annotations in the locus ID v3.30 (Gramene Release 50) database.

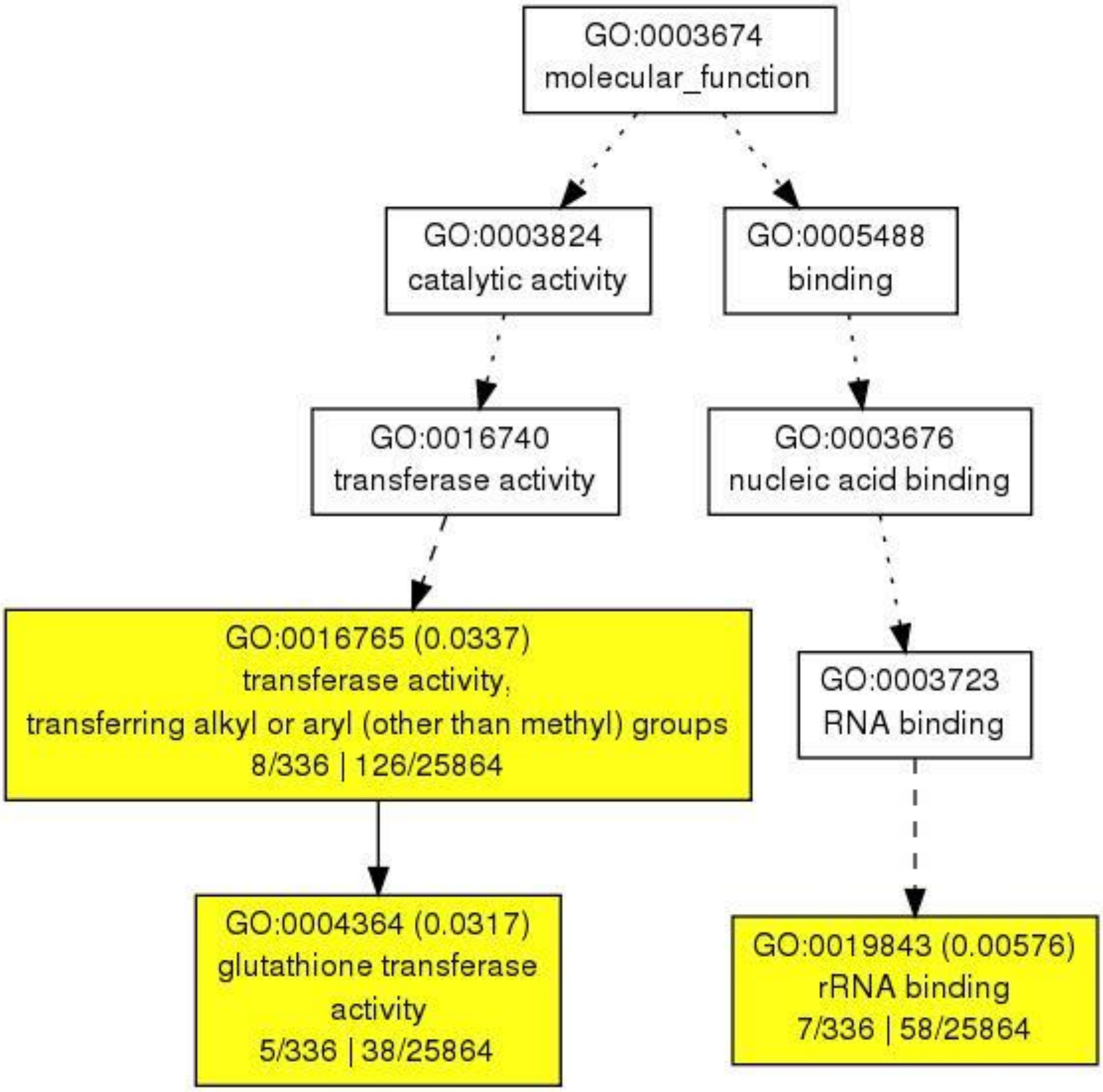

Figure S14. GO analysis carried out on the significantly differentially expressed microarray probes of Control vs LL4 comparison (cold-and light-regulation) regarding to the GO class 'molecular function (MF)'. Overrepresented terms are shown in colored boxes (q < 0.05). Hierarchical graphs show the level of significance of the terms and the linkage among them. Box colors indicate levels of statistical significance: white = parent or non-significant term; yellow = 0.05, brown = 1e−5 and red = 1e-9. Arrow means conjunctions among terms: solid = is a; dashed = two significant nodes; dotted = one significant node, red = positive regulate and green = negative regulate. The pair of numerals in the left represents number of genes in query list associated with that GO term and number of genes in query list. The pair of numerals in the right represents number of genes associated with a particular GO term in the locus ID v3.30 (Gramene Release 50) database and total number of maize genes with GO annotations in the locus ID v3.30 (Gramene Release 50) database.

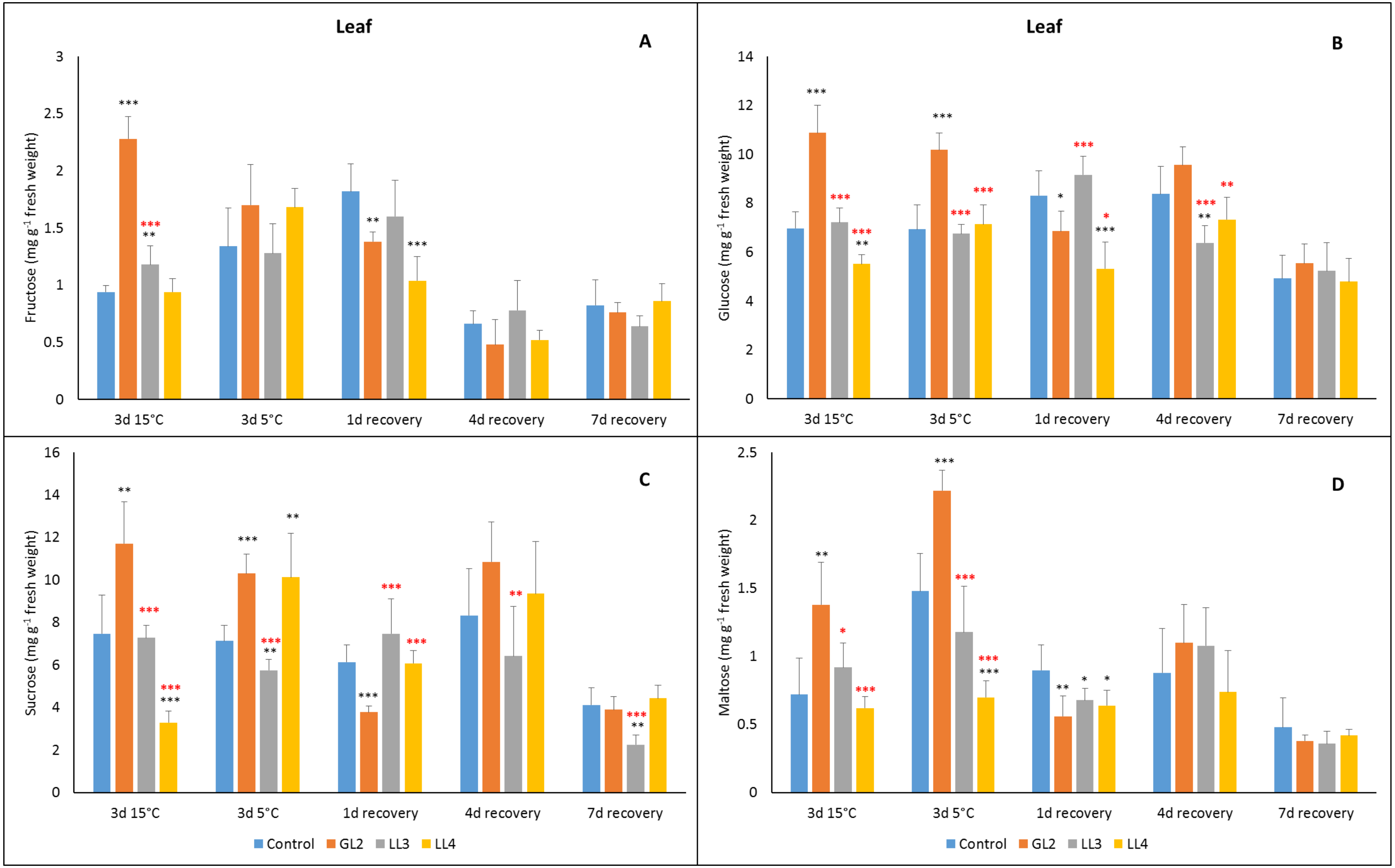

Figure S15. Changes in soluble sugar contents during cold acclimation (15/13 °C), chilling (5°C) and recovery in the leaves of young maize plants. Light intensities during hardening: GL2: 387 µmol m^{-2} s^{-1}; LL3: 283 µmol m^{-2} s^{-1}; LL4: 107 µmol m^{-2} s^{-1}. *, **, *** Significant differences compared to the control plants on the same day at the $p < 0.05$, 0.01 and 0.001 levels, respectively. *, **, *** Significant differences compared to the GL2 plants on the same day at the $p < 0.05$, 0.01 and 0.001 levels, respectively. (A: fructose; B: glucose; C: sucrose; D: maltose).

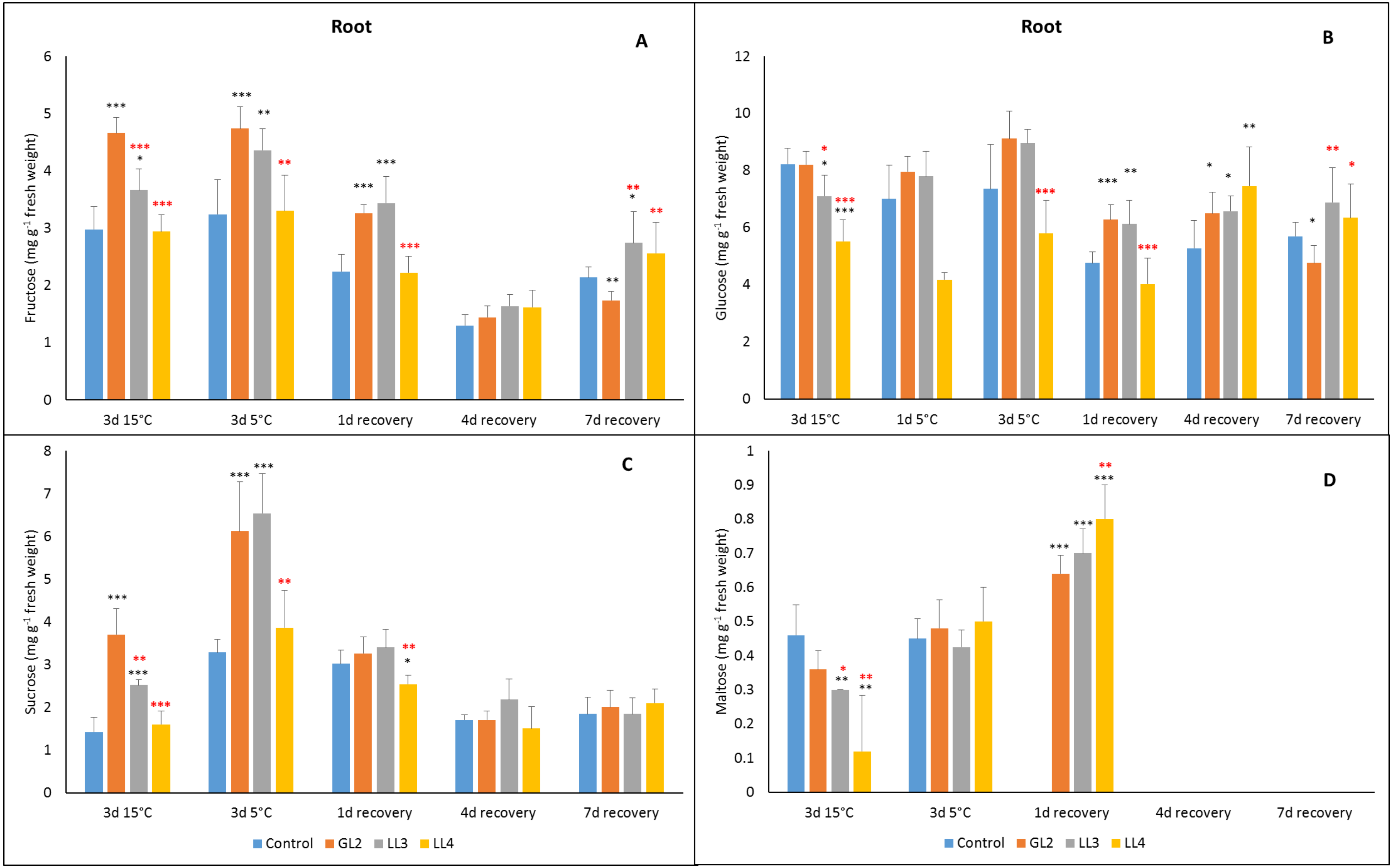

Figure S16. Changes in soluble sugar contents during cold acclimation (15/13 °C), chilling (5°C) and recovery in the roots of young maize plants. Light intensities during hardening: GL2: 387 µmol m^{-2} s^{-1}; LL3: 283 µmol m^{-2} s^{-1}; LL4: 107 µmol m^{-2} s^{-1}. *, **, *** Significant differences compared to the control plants on the same day at the $p < 0.05$, 0.01 and 0.001 levels, respectively. *, **, *** Significant differences compared to the GL2 plants on the same day at the $p < 0.05$, 0.01 and 0.001 levels, respectively. (A: fructose; B: glucose; C: sucrose; D: maltose).
